# Confined Oxidative Polymerization of Preorganized Aniline Within Cyclodextrin-Based Bimolecular Tube Nanochannels for the Synthesis of Bispolypseudorotaxanes

**DOI:** 10.3390/molecules31183353

**Published:** 2026-09-21

**Authors:** Min Deng, Xuze Hu, Yibing Liu, Yuqiang Cheng, Donghua Lai, Lu Huang, Xinli Mou, Junsheng Qi, Changjun Zou

**Affiliations:** 1 Chongqing Key Laboratory of Water Environmental Evolution and Pollution Control in Three Gorges Reservoir, School of Environmental and Chemical Engineering, Chongqing Sanxia University of Science and Technology, Chongqing 404100, China; 19179425752@163.com (M.D.); 13114262356@163.com (Y.L.); 15320721968@163.com (Y.C.); 18759055275@163.com (D.L.); 19822503633@163.com (L.H.); cqsxxymxl@163.com (X.M.); qijunsheng@163.com (J.Q.); 2 Material Corrosion and Protection Key Laboratory of Sichuan Province, Sichuan University of Science & Engineering, Zigong 643000, China; 3 School of Chemistry and Chemical Engineering, Southwest Petroleum University, Chengdu 610500, China; changjunzou@126.com

**Keywords:** CBBT, supramolecular pre-assembly, nanoconfined spaces, PANI/CBBT bispolypseudorotaxanes

## Abstract

The well-defined nanoconfined spaces of cyclodextrin-based bimolecular tubes (CBBTs) make them excellent nanocontainers for directing molecular assembly and polymerization. In this study, we designed and synthesized a novel CBBT with two regular nanochannels. Employed to pre-assemble aniline monomers via host–guest interactions, the CBBT further drove the oxidative polymerization of aniline, thereby forming a polyaniline (PANI)/CBBT bispolypseudorotaxane with specific chain lengths. The structure and morphology of the as-prepared PANI-based bispolypseudorotaxane were characterized using spectroscopic and electron microscopic techniques. The structural features and the host–guest interactions involved were further investigated, demonstrating the potential of this CBBT system as a nanoconfined reaction platform for constructing structurally ordered conjugated polymer materials.

## 1. Introduction

Cyclodextrins (CDs) are cyclic oligosaccharides derived from starch, and their unique hydrophobic cavities have made them indispensable building blocks in carbohydrate-based supramolecular chemistry [1,2,3,4,5,6,7]. Beyond their classical role as molecular hosts for small guests, the covalent or non-covalent assembly of multiple CD units into higher-order architectures such as molecular tubes, channels, and polyrotaxanes has emerged as a powerful strategy to create functional carbohydrate materials with well-defined nanoscale topologies [8,9,10,11,12,13,14,15]. Among these, CD-based molecular tubes (CBMTs), formed by the alignment and crosslinking of two CD molecules, are particularly intriguing because they feature one or two parallel, continuous nanochannels that could serve as nanoreactors for confined chemical transformations [16,17,18,19]. In principle, this channel architecture could provide a unique confined microenvironment that restricts the conformational freedom of encapsulated monomers and directs the course of polymerization, thereby enabling the synthesis of polymer pseudorotaxanes with unprecedented structural precision. These architectures, where cyclic molecules are threaded onto a linear guest molecule without bulky stoppers, exhibit unique dynamic properties and stimuli-responsiveness, making them promising candidates for molecular machines, switches, and adaptive materials [20,21,22,23].

The underlying hypothesis of this work is that aniline monomers can be effectively pre-assembled within the nanochannels of CBBTs via host–guest interactions, and that this pre-assembly, once established, enables the subsequent in situ oxidative polymerization to proceed in a confined manner, directly yielding a PANI/CBBT bispolypseudorotaxane with a defined threaded topology [24]. This comprehensive hypothesis is based on three interconnected premises: (i) the use of a templating method for the efficient preparation of CBBTs; (ii) the pre-assembly of aniline within the CBBT channels is a critical prerequisite that positions the monomer molecules in a spatially organized manner prior to oxidation; and (iii) the nanoconfined space of the CBBT channels constrains the conformational freedom of the growing PANI chains, preventing their escape and ensuring a stable bispolypseudorotaxane architecture [25,26,27]. If validated, this hypothesis would establish CBBTs as versatile carbohydrate nanoreactors for confined polymerization and offer a rational route to well-defined bispolypseudorotaxane materials.

However, this hypothesis faces three major challenges that have hindered progress in this field. First, the synthesis of CBMTs, especially the CBBT, suffers from complicated procedures, low yields, and the requirement of expensive or toxic reagents, making gram-scale or larger-scale production challenging, which severely limits their practical application [28]. Second, the verification of channel size, channel number, and structural regularity of CBBTs is inherently difficult [29]. Third, it remains unclear whether the pre-assembly of aniline by CBBTs can be preserved during the oxidative polymerization under strongly acidic aqueous conditions [30]. The polarity, pH, and competitive hydration in the aqueous polymerization medium could substantially weaken or even disrupt the host–guest association, thereby compromising the presumed preorganization that is essential to the hypothesis [31].

In this work, we present a comprehensive investigation encompassing the preparation, characterization, and nanoconfinement behavior of a CBBT. The dual-channel architecture was quantitatively elucidated by combined WAXS and HR-TEM, which enabled the unambiguous determination of channel size and structural regularity. The host–guest pre-assembly of aniline within the CBBT nanochannels was further probed by UV–vis and 2D NMR spectroscopy. Subsequently, in situ oxidative polymerization of aniline was conducted within the nanoconfined space, and the resulting PANI/CBBT bispolypseudorotaxane was systematically evaluated with respect to its structural features, morphological characteristics, and stability profile.

## 2. Results and Discussion

### 2.1. Synthesis of CBBT and Its Structure

**Scheme 1:**
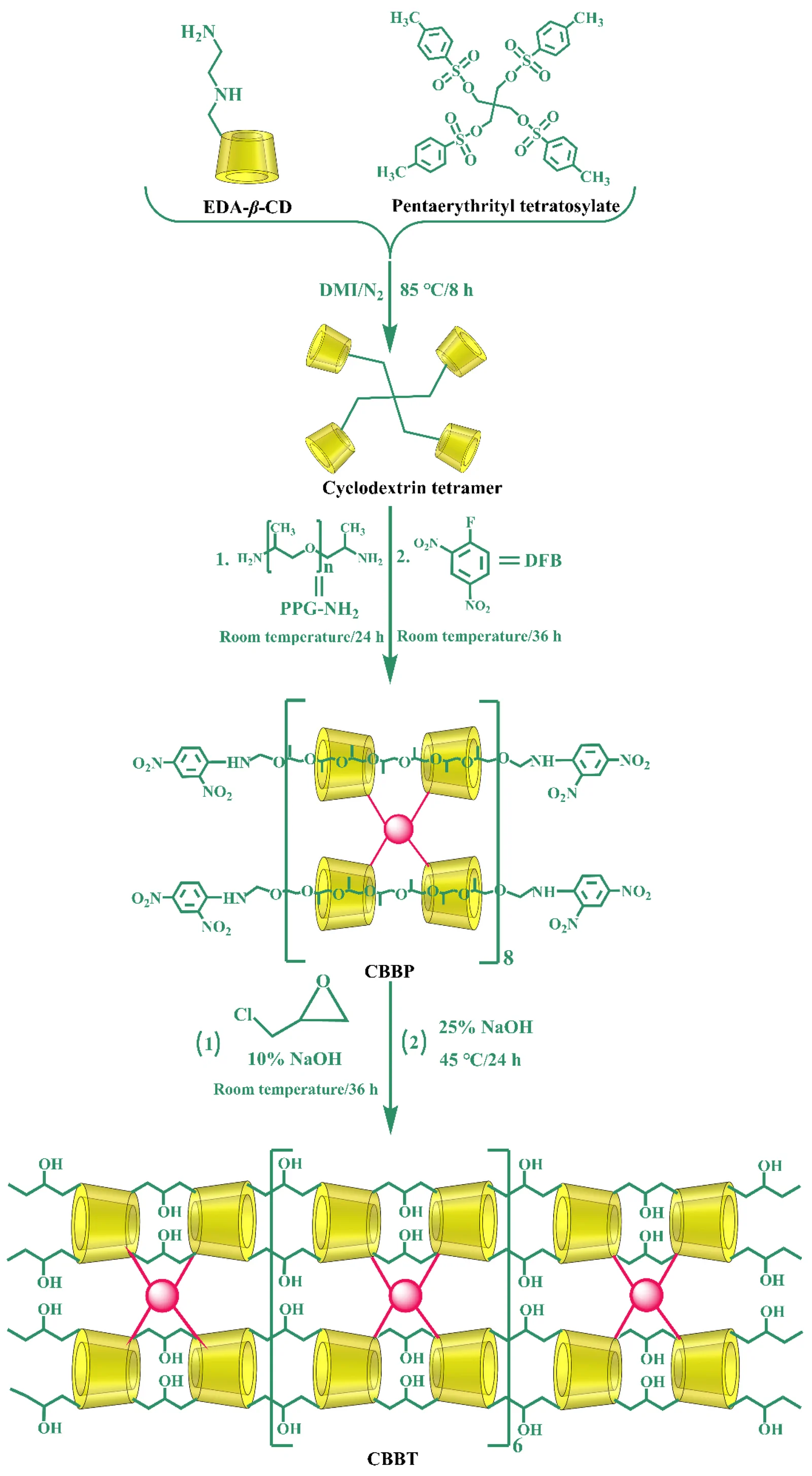
Synthetic route to the PANI/CBBT bispolypseudorotaxane.

The CBBT was prepared according to Scheme 1. The initial step was the synthesis of a cyclodextrin tetramer through nucleophilic substitution of pentaerythrityl tetratosylate with EDA-β-CD. From then on, a preorganized bisrotaxane was first prepared by threading PPG-NH_2_ chains through a cyclodextrin tetramer, and the subsequent capping reaction of this bispseudorotaxane with DFB in aqueous medium. In the final step, CBBTs were synthesized by crosslinking adjacent β-CD rings in the bisrotaxane with epichlorohydrin, followed by cleavage of the terminal blocking groups and removal of the polymeric axle under alkaline conditions. Prior to the discussion of the CBBT structure, the characterization results of its synthetic precursors are provided in the Supporting Information. As shown in Figures S1–S18, the spectroscopic data for pentaerythrityl tetratosylate, EDA-β-CD, cyclodextrin tetramer, and CBBP are systematically documented, confirming the successful preparation of each intermediate.

**Figure 1:**
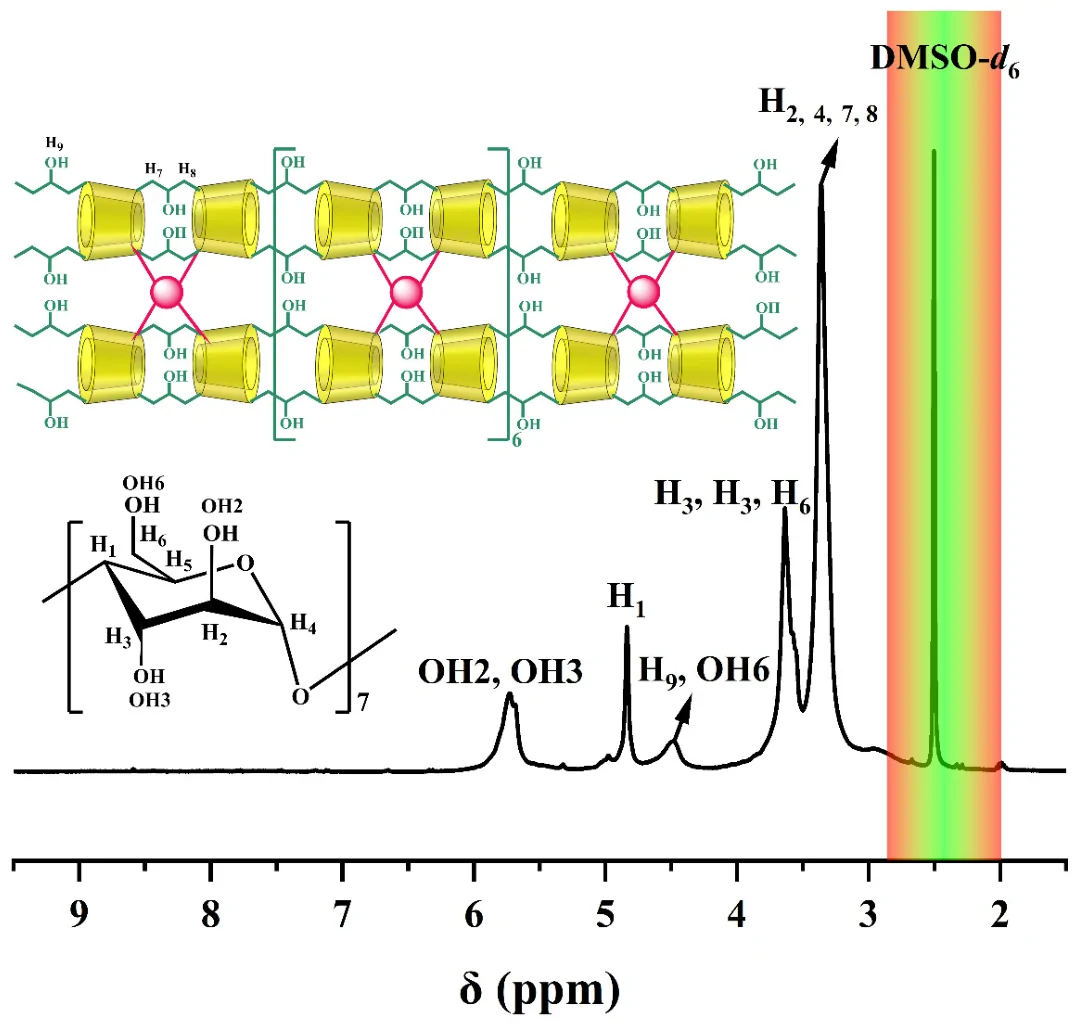
^1^H NMR spectra of CBBT in DMSO-*d*_6_.

The structure of the proposed CBBT is shown in Scheme 1. To confirm the formation of the CBBT, its structure was characterized via FT-IR and ^1^H NMR spectroscopic methods. In the FT-IR (Figure S19) spectra of the CBBT, there is the formation of a new peak located at 1428 cm^−1^, which might be attributed to the hydroxypropyl (-CH_2_-CH(OH)-CH_2_-) group. The characteristic peaks at 1157 cm^−1^ and 939 cm^−1^ were ascribed to C-O-C stretching bands from the glycosidic backbone of α-(1, 4)-linkages. Meanwhile, the characteristic FT-IR peaks of the end-capping agent DFB were entirely absent in the spectrum, indicating its complete consumption. SMoreover, the signals observed at 5.73–5.67 and 4.84 (OH2-OH3, H_1_) ppm were assigned to the protons of the β-CD units, respectively, as shown in Figure 1. Signals located within the range of 4.59–4.49 and 3.86–2.98 (OH6, H_2_-H_9_) ppm were attributed to the protons of the β-CD moieties and 2-propanol residues. The successful crosslinking by epichlorohydrin, along with the elimination of the PPG-NH_2_ polymer axle and the end-capping agent, was corroborated by combined FT-IR and ^1^H NMR spectroscopic analyses. The XRD patterns of the TsO-β-CD, EDA-β-CD, cyclodextrin tetramer, CBBP, and CBBT are compared in Figure S20a,b. TsO-β-CD shows numerous sharp diffraction peaks, characteristic of a crystalline phase (Figure S20a). In contrast, the EDA-β-CD, cyclodextrin tetramer, CBBP, and CBBT each exhibit only a single major diffraction peak, indicative of a powder-like state (Figure S20b). Additionally, the main diffraction peak gradually shifts from 18.5° to 18.7°, 18.8°, and 19.1° for the EDA-β-CD, cyclodextrin tetramer, CBBP, and CBBT, respectively, suggesting progressive structural changes in the β-CD framework during the stepwise modification.

**Figure 2:**
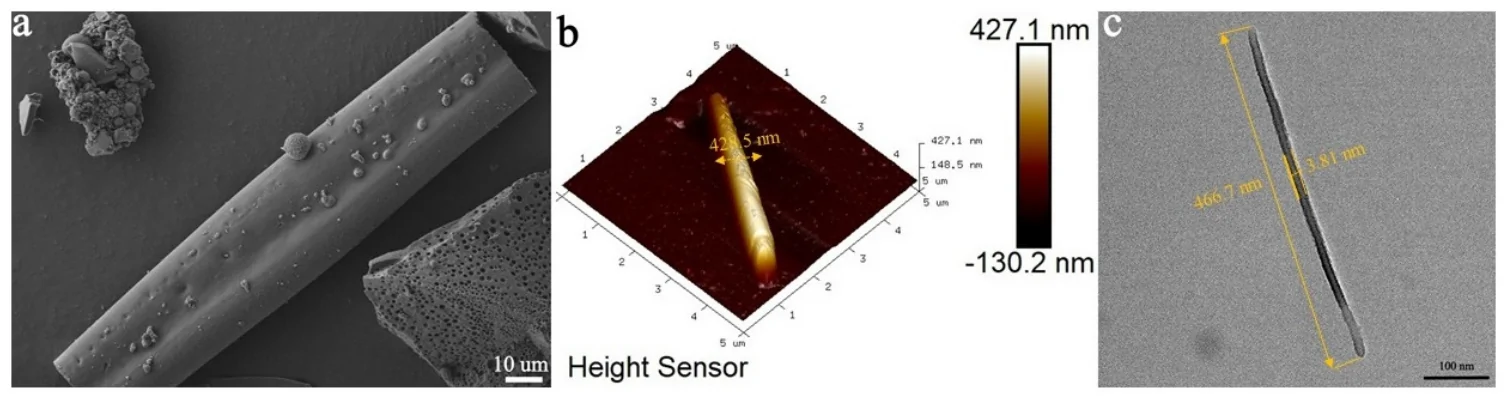
Electron microscope images of CBBT: (**a**) SEM, (**b**) AFM, (**c**) HR-TEM.

The tubular structure of the CBBT was further verified by SEM, AFM, HR-TEM, and WAXS tests, as shown in Figure 2a–c and Figure S21. From the SEM image (Figure 2a), it can be further observed that the CBBT displayed an ordered block-like morphology with a large surface size, which may result from intermolecular crosslinking or intermolecular aggregation. The AFM image of the CBBT more displayed an ordered cylindrical morphology with a width of ~428.5 nm and a height of ~427.1 nm that corresponded to the aggregation of individual assemblies into a rod-like structure owing to the large surface energy (Figure 2b). Analogously, the HR-TEM image gave a rough insight into the size and shape of the CBBT as a linear structure with a length of ~466.7 nm and a width of ~3.81 nm (Figure 2c). The analysis above indicated that the CBBT morphology exhibited a rigid tubular structure. The WAXS pattern of the CBBT is presented in Figure S21. Notably, only one distinct diffraction peak was detected at q = 0.3641Å^−1^, without any higher-order reflections, indicating a powder-like state with limited crystallinity. This finding was in good agreement with the XRD analysis, which similarly suggested the absence of long-range crystalline order. Such a diffraction profile was characteristic of covalently crosslinked polymeric nanotubes, where structural coherence was restricted by the curved geometry and the flexible nature of the bridging segments. Despite the limited number of diffraction peaks, the observed reflection enables the calculation of the tubular cross-sectional dimension. Using the Bragg-like relationship d = 2π/q, the tubular diameter was calculated to be ~3.451 nm. This value corresponds well with the HR-TEM observations, which directly visualize a tubular architecture with comparable dimensions. The close match between the WAXS-derived diameter and the HR-TEM measurement strongly supports the assignment of the diffraction peak to the cross-sectional periodicity of the CBBT double-row tubules, rather than to cyclodextrin assemblies/aggregates.

### 2.2. Pre-Assembly-Mediated Oxidative Polymerization of Aniline

**Scheme 2:**
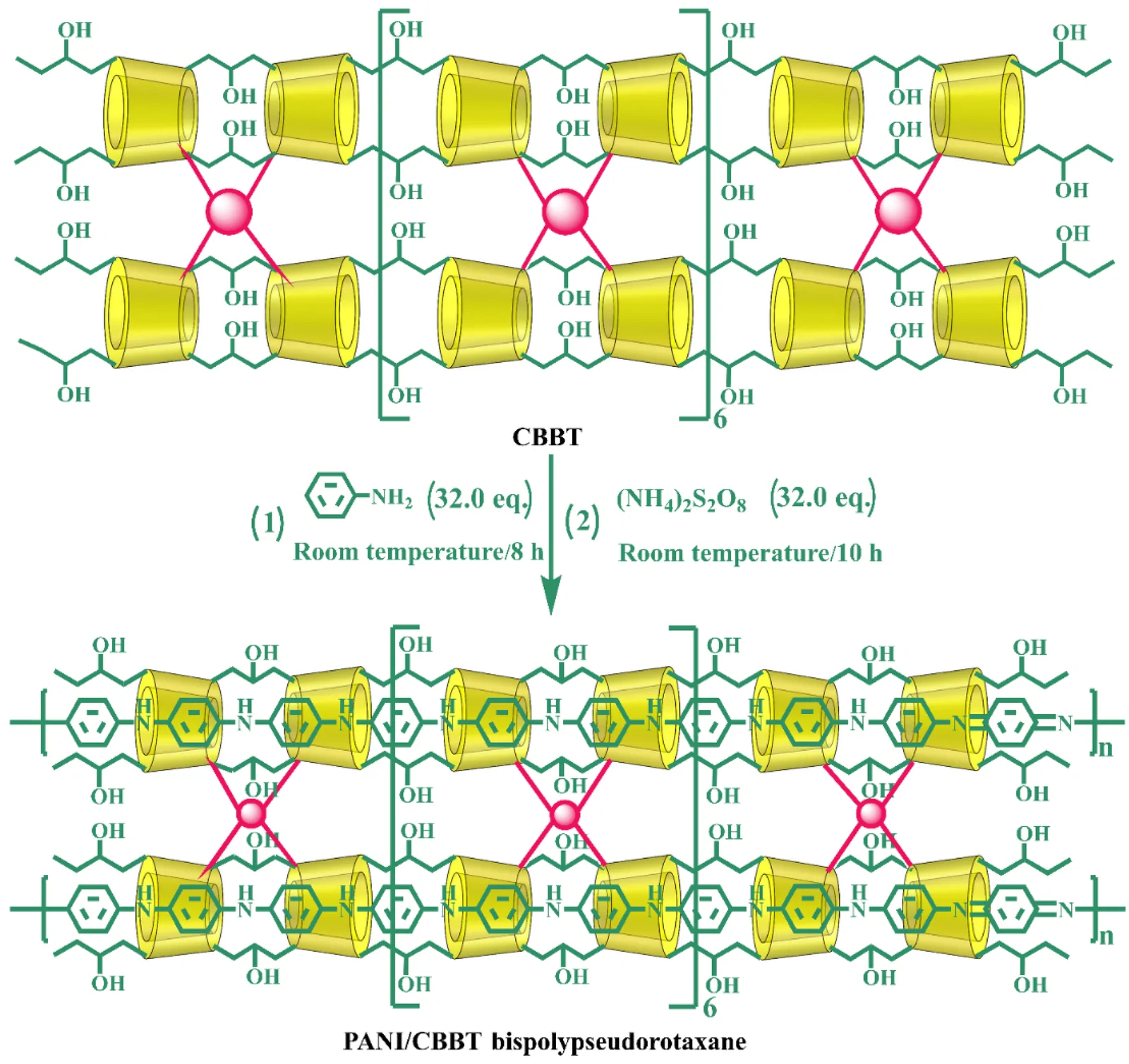
Synthetic route of PANI/CBBT bispolypseudorotaxane.

The PANI/CBBT bispolypseudorotaxane was prepared according to Scheme 2. The CBBT can drive aniline into its regular cavities via supramolecular host–guest interactions to form a bispseudorotaxane precursor, which was subsequently converted into a PANI/CBBT bispolypseudorotaxane through direct oxidation and threading assembly.

**Figure 3:**
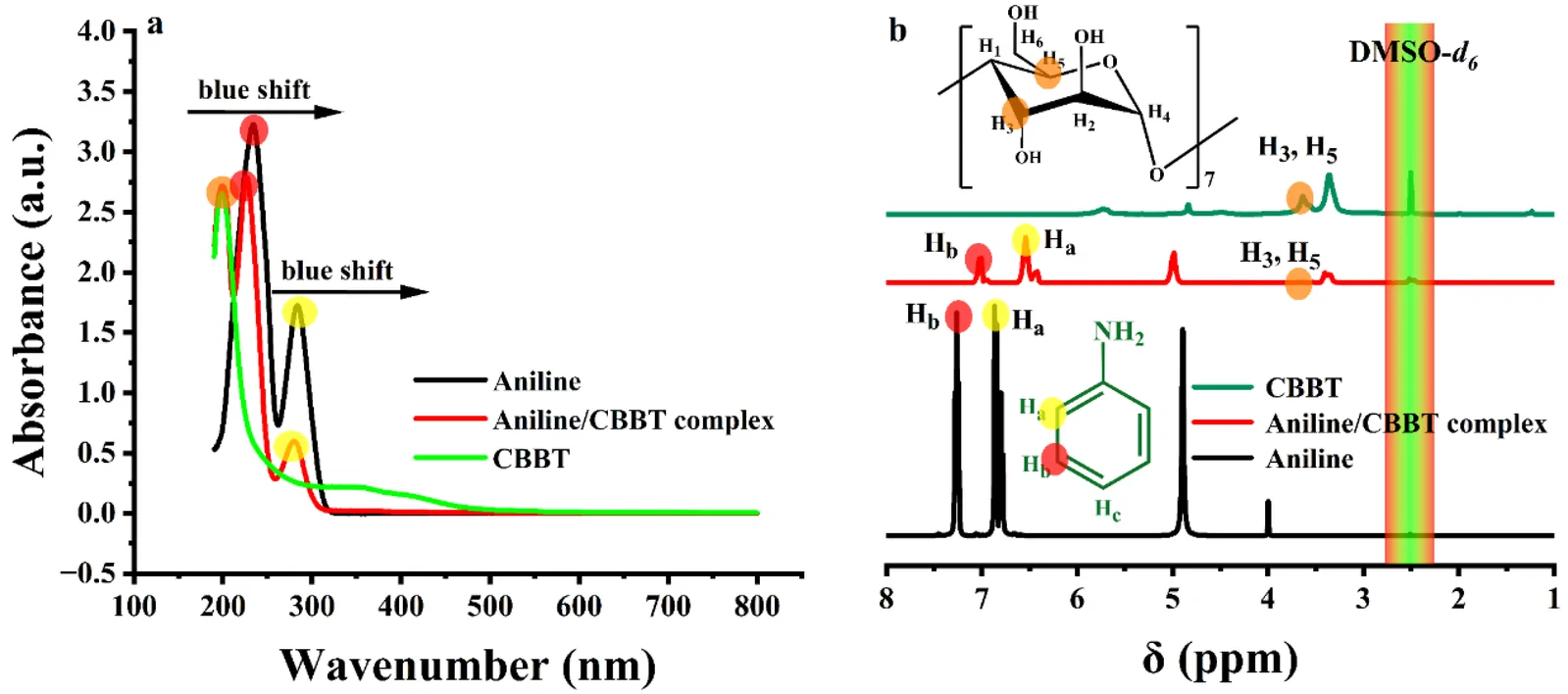
(**a**) The UV–vis spectra of aniline, CBBT, and aniline/CBBT complex. (**b**) ^1^H NMR spectra of aniline, aniline–CBBT complex, and CBBT in DMSO-*d*_6_.

**Figure 4:**
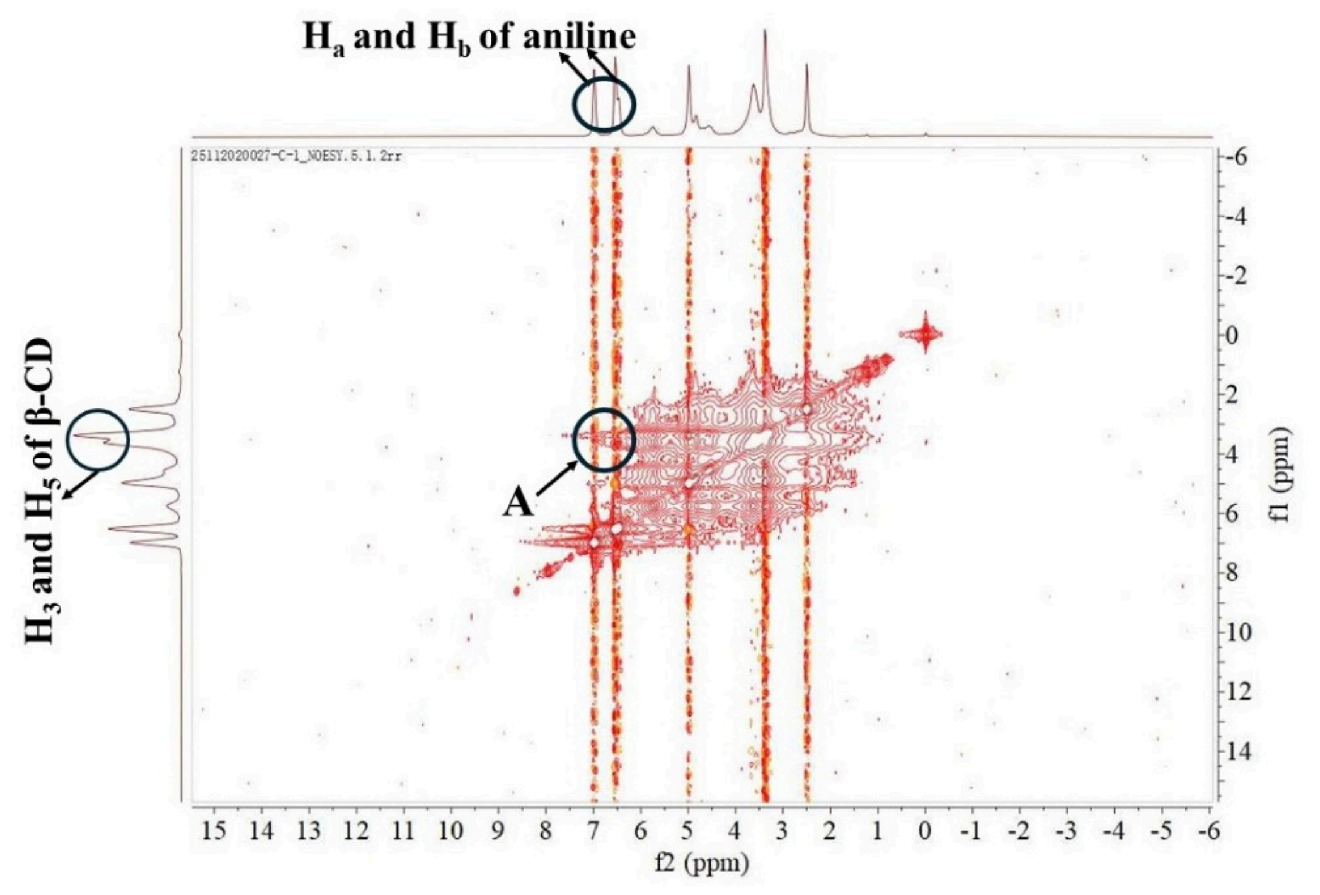
Sectional 2D ROESY NMR spectrum of aniline (5 μL) and CBBT (60 mg) with a mixing time of 5 h in DMSO-*d*_6_.

In the initial step, aniline undergoes protonation in the presence of hydrochloric acid, forming anilinium cations (C_6_H_5_NH_3_^+^). This protonation process is essential for rendering aniline soluble in the aqueous phase and providing the positively charged species required for subsequent oxidative polymerization. Importantly, the cyclodextrin cavity exerts a stabilizing effect on the anilinium cation intermediates during the oxidation process. This stabilization arises from the host–guest interactions between the hydrophobic cavity of cyclodextrin and the encapsulated anilinium species. By sequestering the reactive intermediates within the confined microenvironment, the cyclodextrin cavities prevent their premature aggregation or uncontrolled precipitation, which are commonly observed in conventional bulk polymerization. Consequently, the oxidative coupling and chain propagation steps proceed in a more controlled and spatially constrained manner within the CBBT channels. Compared with conventional bulk polymerization—where anilinium cations and oxidative intermediates are free to diffuse and aggregate randomly in solution—the confinement within CBBT channels imposes significant spatial constraints on the polymerization process. The encapsulation and stabilization of anilinium cations within the cyclodextrin cavities lead to a template growth mechanism, wherein the polymer chains propagate along the confined nanospace defined by the tubular channels. This confined environment promotes a more orderly chain propagation and nucleation process, ultimately yielding polyaniline nanostructures with well-defined morphologies that are distinct from those obtained by bulk polymerization. In essence, the CBBT channels serve as nanoreactors that direct the polymerization pathway through a combination of host–guest stabilization and spatial confinement. The pre-assembly-driven polymerization behavior of aniline within the CBBT was monitored by UV–vis and the NMR technique, as presented in Figure 3a,b and Figure 4, and S22. A distinct blue shift (shifted downfield) was detected in the characteristic UV–vis absorption peaks (at 226 nm and 275 nm) for the aniline/CBBT bispseudorotaxane precursor compared to pure aniline (at 230 nm and 280 nm) (Figure 3a). This can be primarily attributed to the stronger stabilization of the excited state by polar solvents, while the energy gap between the ground and excited states widens in a nonpolar environment, resulting in a blue shift in the absorption wavelength. Therefore, the blue shift observed in the UV spectrum confirmed the access of aniline into a more hydrophobic environment—namely, the interior cavity of β-CD. For more detailed pre-assembly interaction between aniline and the CBBT, ^1^H NMR analysis revealed a downfield shift of Δδ = 0.32–0.25 ppm for the aniline protons H_a_/H_b_ based on the amplifying treatment (Figure S22), while the H_3_/H_5_ protons of the β-CD fragments in the CBBT shifted upfield by Δδ = 0.02 ppm (Figure 3b), respectively. However, the possibility existed that this signal originated from intramolecular interactions within the aniline molecule. In the 2D ROESY NMR spectra (Figure 4), a pronounced cross-peak (labeled A) was detected, which indicated a correlation between the β-CD moiety protons (H_3_, H_5_) and the aniline protons (H_a_, H_b_). Based on the above results, aniline is encapsulated within the nanoconfined cavity of CBBT via host–guest interactions, leading to the formation of a pre-assembled aniline/CBBT bispseudorotaxane precursor.

**Figure 5:**
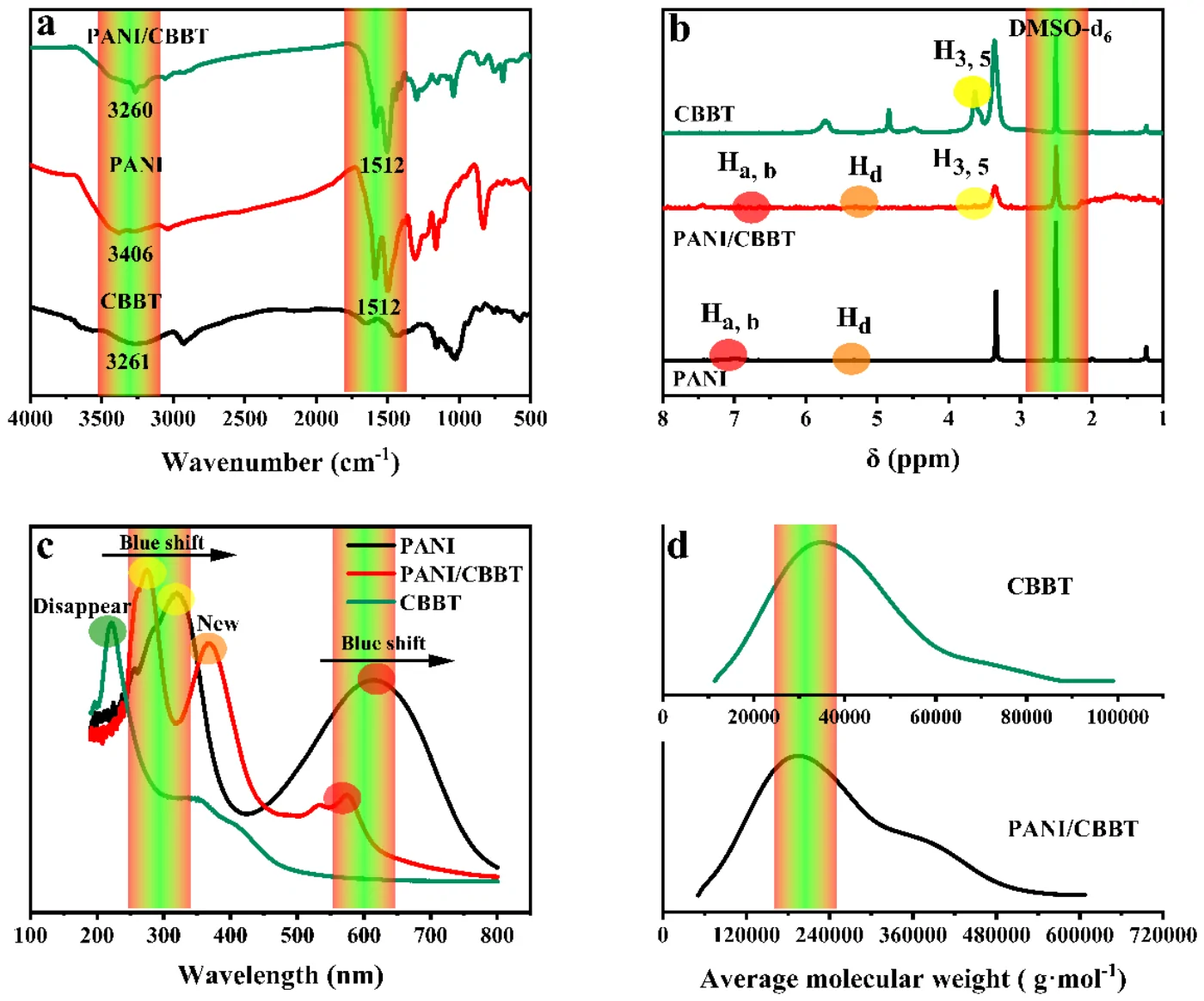
Spectroscopic characterization of PANI/CBBT bispolypseudorotaxane. (**a**) FT-IR spectra. (**b**) ^1^H NMR spectra. (**c**) UV–vis spectra. (**d**) Average molecular weight curves.

**Figure 6:**
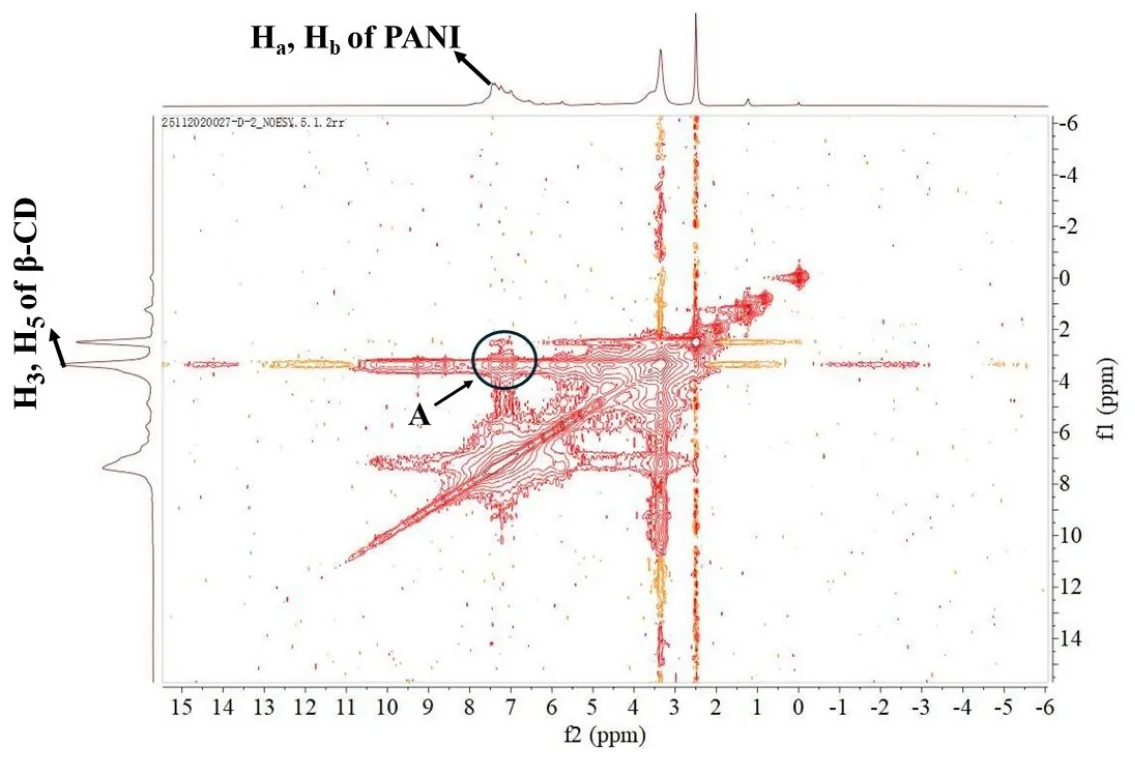
2D ROESY NMR spectrum of PANI/CBBT bispolypseudorotaxane (50 mg) with a mixing time of 5 min in DMSO-*d*_6_.

Pre-assembly-mediated oxidative polymerization of aniline was studied using FT-IR, UV–vis, NMR, and XRD spectroscopic methods, as shown in Figure 5a–c, Figures S21a–c and S22. By comparing the FT-IR spectra of the CBBT and PANI (Figure 5a), a new peak located at 1512 cm^−1^ is observed, which might be attributed to the C=C (benzene ring) bond from PANI. The proton signals of ^1^H NMR at 7.21/7.03 ppm (H_a_, H_b_) and 5.31 ppm (H_d_) in free PANI are ascribed to the doped phenyl and quinoid structural units, respectively (Figure 5b). An upfield shift was observed for the benzene ring protons (H_a_, H_b_) of the aniline moiety, while the chemical shifts in the quinoid protons (H_d_) in PANI and the inner-cavity protons (H_3_, H_5_) of the β-CD units all exhibited a downfield shift via careful observation based on the amplifying treatment in the PANI/CBBT bispolypseudorotaxane (Figure S21a–c). Accordingly, a strong cross-peak (labeled A) was observed in the 2D NMR spectrum, indicating a correlation between the H_a_/H_b_ protons of the PANI chain and the H_3_/H_5_ protons of the β-CD segments (Figure 6). Meanwhile, in the UV–vis spectra of the PANI/CBBT bispolypseudorotaxane (Figure 5c), the characteristic peaks of PANI at 320 nm and 620 nm displayed distinct blue shifts to 274 nm and 576 nm, which could originate from nonspecific interactions such as surface adsorption or blending. Of note, the characteristic peak of the CBBT at 221 nm disappeared, while a new peak occurred at 368 nm. Upon oxidative polymerization, in contrast to the conventional polymerization of free aniline, the resulting PANI chains threaded through the CBBT macrocycles, thereby affording PANI/CBBT bispolypseudorotaxanes.

Compared with the XRD pattern of the pure PANI, the as-prepared PANI/CBBT bispolypseudorotaxane exhibited multiple broadened and attenuated diffraction peaks, indicating a reduction in the crystallinity of these samples (Figure S22). The average molecular weight of the CBBT and PANI/CBBT bispolypseudorotaxane was performed and analyzed by GPC, as shown in Figure 5d. A unimodal molecular weight distribution was observed in the GPC curves, indicating that no homopolymers existed in the CBBT and PANI/CBBT bispolypseudorotaxane. Accordingly, the average molecular weight of the CBBT and PANI/CBBT bispolypseudorotaxane was measured to be ~3.8 × 10^4^ g·mol^−1^ and ~2.0 × 10^5^ g·mol^−1^, respectively. Analysis of the average molecular weights of the CBBT indicated an average degree of polymerization of approximately 8 for the cyclodextrin tetramer units, which was consistent with a homogeneous polymeric structure rather than polydisperse aggregates. Moreover, the molecular weight analysis of the resulting PANI/CBBT bispolypseudorotaxane demonstrated that aniline polymerization was directionally confined along the axial orientation of the molecular tube inside the CBBT nanoconfined spaces.

**Figure 7:**
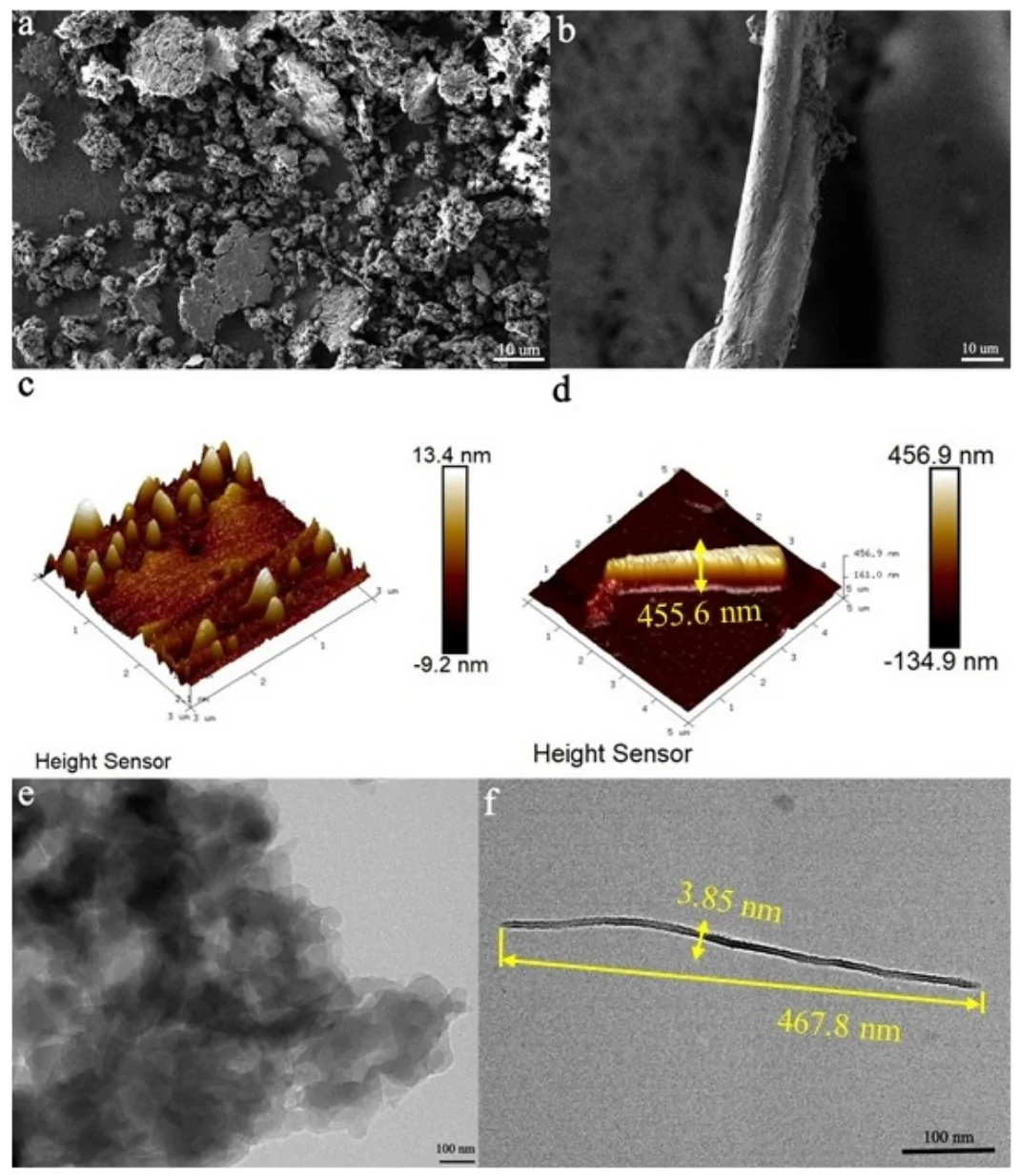
Electron microscope images of PANI and PANI/CBBT bispolypseudorotaxane: (**a**,**b**) SEM, (**c**,**d**) AFM, and (**e**,**f**) HR-TEM.

The supramolecular assembly-mediated morphological modulation of PANI was investigated by SEM, AFM, and HR-TEM techniques, as shown in Figure 7a–f. As shown in the SEM images (Figure 7a), PANI polymerized in the absence of the CBBT exhibited an irregular morphology. Significantly, the oxidative polymerization of aniline pre-assembled within the CBBT resulted in a PANI/CBBT bispolypseudorotaxane with a uniform cylindrical shape (Figure 7b). This distinct architecture underscores the directed growth of PANI within the confining channels of the CBBT template. Correspondingly, the AFM images revealed distinct topographies where PANI appeared as irregular particles (Figure 7c). In contrast, the PANI/CBBT bispolypseudorotaxanes exhibited ordered cylindrical morphologies (Figure 7d). The dimensions were notably similar: the bispolypseudorotaxane has a width of ~455.6 nm and an average height of ~456.9 nm, closely matching the dimensions of the CBBT itself (~427.1 nm in height). Similarly, the HR-TEM images (Figure 7e) showed that PANI possessed a disordered morphology. Notably, the PANI/CBBT bispolypseudorotaxanes exhibited well-defined columnar structures (Figure 7f). Their dimensions were remarkably similar: the CBBT measured ~466.7 nm in length and ~3.81 nm in width, while the bispolypseudorotaxane was ~467.8 nm long and ~3.85 nm wide. The substantial difference between the AFM-measured diameter (455.6 nm) and the HR-TEM-measured diameter (3.85 nm) warrants further discussion. AFM measurements were typically performed on dried samples, where the CBBT templates tend to aggregate or bundle together due to capillary forces and intermolecular interactions during the drying process. Consequently, the AFM-measured diameter reflects the overall dimensions of aggregated CBBT bundles rather than the intrinsic diameter of individual tubular channels. In contrast, HR-TEM imaging enables direct visualization of individual nanostructures with minimal interference from aggregation, thus providing a more accurate representation of the intrinsic channel dimensions. The close agreement between HR-TEM (3.85 nm) and WAXS (3.451 nm) confirmed that the HR-TEM measurement accurately reflects the intrinsic tubular diameter of the CBBT. Meanwhile, the close agreement between HR-TEM (3.85 nm) and WAXS (3.451 nm) confirmed that the polymerization occurred within the confined nanochannels of the CBBT, while the larger AFM diameter reflects template aggregation rather than intrinsic channel dimensions. These combined results clearly demonstrate that the observed columnar morphology was generated by the CBBT template and was not merely a result of PANI/CBBT aggregation. Together with the SEM and AFM analyses, these results collectively demonstrated that the addition of the CBBT can effectively regulate the morphology of the PANI-based bispolypseudorotaxanes.

**Figure 8:**
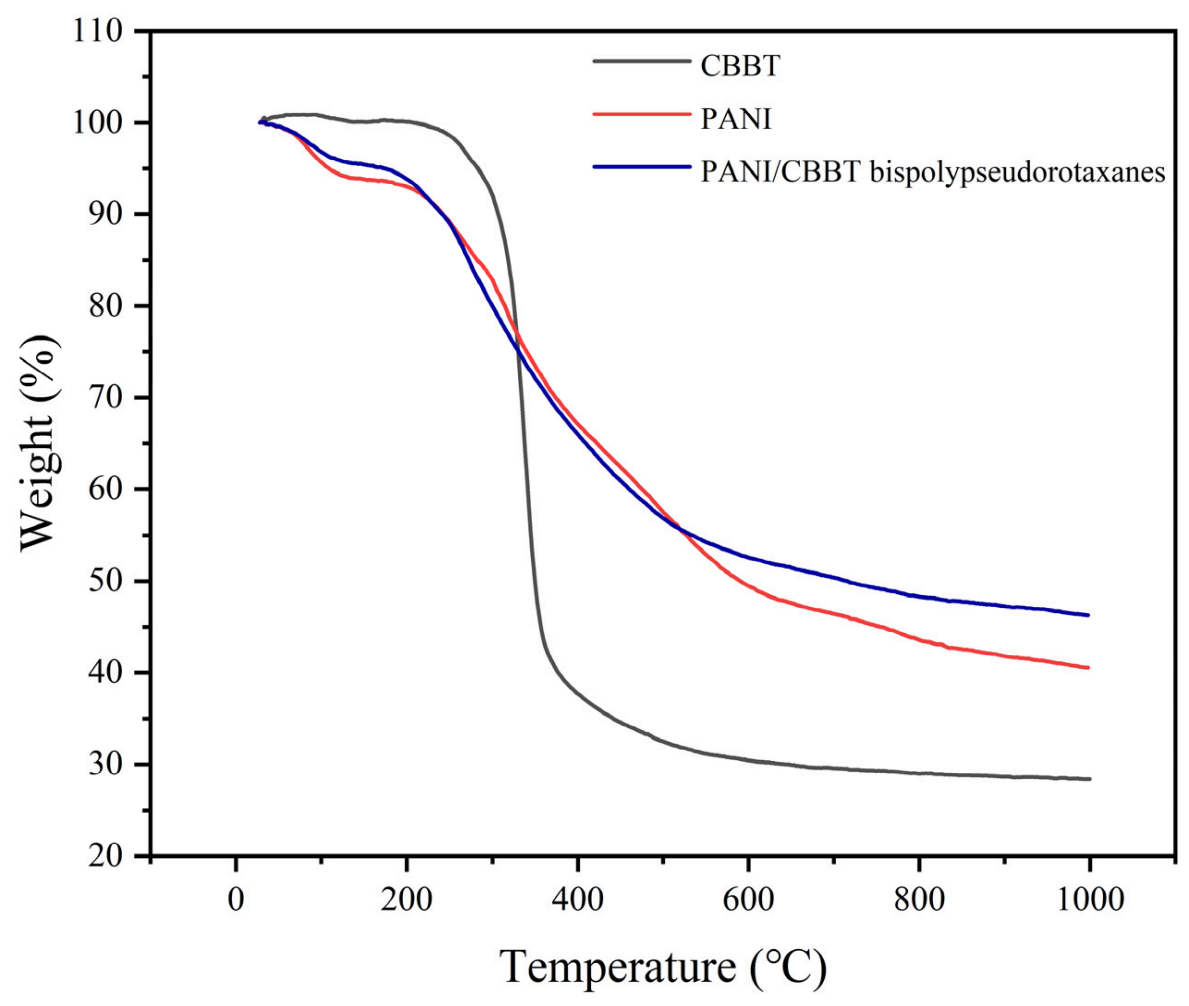
TGA curves of CBBT, PANI and PANI/CBBT bispolypseudorotaxanes.

The thermal stability of the supramolecular assembly was evaluated via TGA, with the mass retention values shown in Figure 8. All specimens exhibit negligible mass loss below 200 °C, indicative of efficient removal of physiosorbed water and residual solvents without compromising the supramolecular scaffold. Pristine PANI underwent a rapid two-stage degradation between 206 °C and 627 °C, attributable to the deprotonation of the emeraldine salt and subsequent scission of the quinoid–benzenoid backbone, leaving a carbonaceous residue of merely 40.3% at 1000 °C. Above 240 °C, the CBBT displayed a steep mass decline, culminating in a 28.4% char residue at 1000 °C. For the threaded bispolypseudorotaxanes, the degradation profile exhibited a distinct sheltered behavior throughout the primary decomposition stage (200–650 °C). Notably, at 206 °C and 627 °C, the composite retains 94.2% and 52.1% mass, respectively, compared to 92.1% and 48.5% for PANI under identical conditions. The threaded PANI chains, physically confined within the CBBT cavities, encountered a substantial steric barrier that impeded the outward diffusion of volatile oligomeric fragments. Moreover, the extensive hydrogen-bonding network between the polyaniline –NH– groups and the cyclodextrin secondary hydroxyls effectively raised the activation barrier for the initial unzipping of the PANI chain. This confinement stabilization effect was particularly evident at 627 °C, where the composite retains 52.1% mass versus 48.5% for neat PANI, implying that the CBBT acts as a transient thermal shield. Nevertheless, at temperatures exceeding 700 °C, the three curves moved toward residual values (28.4, 40.3, and 46.2% at 1000 °C, respectively), suggesting that the ultimate char formation was thermodynamically governed by the intrinsic aromaticity of the system, with the CBBT component exerting a dominant influence on the final residue. These data collectively demonstrated that mechanical interlocking in the bispolypseudorotaxanes effectively decelerated the early-stage pyrolytic chain scission without altering the fundamental decomposition chemistry of either subcomponent.

## 3. Materials and Methods

### 3.1. Materials

Mono-6-O-(p-toluenesulfonyl)-β-cyclodextrin (TsO-β-CD, >95.0%, HPLC, CAS:67217-55-4), pentaerythritol (≥99%, T, CAS:115-77-5), poly (propylene glycol) bis (2-aminopropyl ether) (PPG-NH_2_, average molecular weight ~2000, AR, CAS: 9046-10-0), ethylenediamine (>98.0%, T, CAS:6780-13-8), epichlorohydrin (>99.0%, GC, CAS:106-89-8), 2, 4-dinitrofluorobenzene (DFB, >99.0%, GC, CAS:70-34-8), sodium hydroxide (NaOH, >98.0%, T, CAS:1310-73-2), hydrochloric acid (HCl, ≥37.4%, AR, CAS: 7647-01-0), nitric acid (HNO_3_, 70%, ACS, CAS:7697-37-2), 1, 3-dimethyl-2-imidazolidinone (DMI, >99.0%, GC, CAS:80-73-9), N-Methyl pyrrolidone (NMP, ≥99%, ACS, CAS:872-50-4), and ethanol (≥99.5%, ACS, CAS:64-17-5) were purchased from Aladdin Biochemical Technology Co., Ltd. Shanghai, China. All chemical reagents were used as received without further purification.

Fourier transform infrared spectroscopy (FT-IR) spectra of prepared samples were recorded on a Nexus 670 instrument (Thermo Nicolet, USA). Transmittance measurements were conducted in the wavelength range 500–4000 cm^−1^ and 16 scans per sample under room temperature and atmosphere pressure.

Nuclear magnetic resonance spectroscopy (NMR) measurements were recorded on a Bruker AVL 400 MHz spectrometer (Bruker, Germany) using DMSO-*d*_6_ and D_2_O as solvents at room temperature. The chemical shifts are reported as δ values (ppm) relative to the solvent residual peak (DMSO-*d*_6_: 2.52 ppm for ^1^H and 39.6 ppm for ^13^C; D_2_O: 4.80 ppm for ^1^H).

Mass spectrometric analyses were performed on a Bruker ultraflextreme MALDI-TOF/TOF mass spectrometer equipped with a Smartbeam-II laser (355 nm) operating at a repetition rate of 2 kHz. The instrument was externally calibrated in positive reflector mode using the Peptide Calibration Standard II prior to the analysis of samples.

Powder X-ray diffraction (XRD) patterns were acquired using a Bruker D8 diffractometer (Bruker AXS, Germany) with Cu Kα radiation (λ = 1.542 Å), and the scanning rate was 2°/min from 10° to 70° (2θ) under a 3 kW power. The q vector values were calculated according to the equation: q = 4πsinθ/λ.

WAXS measurements were performed on a Xeuss 3.0 SAXS/WAXS system (Xenocs, France) equipped with a Cu Kα X-ray source (8.05 keV, λ = 1.54189 Å) and a DECTRIS Eiger2R 1M hybrid pixel detector with a pixel size of 75 µm × 75 µm. The sample-to-detector distance was calibrated using a standard reference material (e.g., silver behenate) prior to data collection. The scattering patterns were recorded under vacuum conditions (<1 mbar) to minimize air scattering. To quantitatively evaluate the structural dimensions of the CBBT double-row tubules, the WAXS patterns were analyzed using the scattering vector formalism. For each diffraction peak, the scattering vector modulus was defined as follows:

q = (4πsinθ)/λ(1)

where λ = 1.54189 Å (Cu Kα radiation) and 2θ is the full scattering angle. In the WAXS pattern, the most intense diffraction peak in the low-angle region was assigned to the cross-sectional periodicity of the tubular structure. According to the Bragg condition generalized for small-angle/wide-angle scattering, the real-space periodic distance d is inversely proportional to the scattering vector:

d = 2π/q(2)

where q corresponds to the peak maximum position in the q-space profile. This equation arises from the Fourier transform relationship between real-space electron density distribution and reciprocal-space scattering intensity. The calculated d value thus provides a direct measure of the average center-to-center distance between the two parallel channels in the CBBT double-row tubular framework. All reported diameter values were obtained from at least three independent measurements and are presented as mean ± standard deviation.

Morphological characterization of the prepared samples was done using a scanning electron microscope (SEM, Hitachi Regulus8100), high-resolution transmission electron microscope (HR-TEM, FEI Tecnai F20), and atomic force microscope (AFM, Bruker Dimension Icon). The samples for the SEM observations were prepared by depositing several drops of the solution (1 mg/mL) onto the surface of cleaned glass. After excess solution was removed with a spin coater, the samples were dried at room temperature for 24 h. The samples were coated with a thin film of gold before measuring. The HR-TEM samples were prepared by placing a drop of the aqueous solution of the samples in the concentration of 2 × 10^6^ M onto a carbon-coated copper grid, and the excess solution was removed with a strip of filter paper after 5 min. The samples were examined by an HR-TEM operating at an accelerating voltage of 200 kv. For the AFM measurements, the preparation of the samples was similar to that of the SEM experiments except that the fresh cleaved mica was used as stages and gold coating was omitted. A freshly cleaved mic surface was used as a substrate without surface treatment.

The average molecular weight of the CBBT was determined by a gel permeation chromatography (GPC, PL-GPC50) system at 40 °C with water as the mobile phase at a flow rate of 0.6 mL/min. Detection was performed with a diode array detector at a detection wavelength of 205 nm. Both polystyrene and poly(ethylene oxide) were used for calibration.

Thermogravimetric analysis (TGA) was carried out on a Netzsch STA 449 F5 simultaneous thermal analyzer equipped with a cooling system. Samples weighing 5–10 mg were placed in Al_2_O_3_ crucibles and heated from 30 °C to 1000 °C at a heating rate of 10 °C/min under a nitrogen flow of 25 mL/min.

### 3.2. Synthesis of Pentaerythrityl Tetratosylate

A total of 3.94 g (0.029 moL) of pentaerythritol was dissolved into dry pyridine (35 mL) in a three-necked flask under stirring. Then, p-toluenesulfonyl chloride (22.17 g, 0.116 moL) was dropwise added into the reaction mixture and vigorously stirred for about 48 h at 0–5 °C. The reaction was terminated and iced water was added. The precipitate was collected by filtration and washed with deionized water. (KBr, cm^−1^): 3200–3400 (O-H, stretching), 3055 (aromatic C-H, stretching), 1598 and 1458 (para-disubstituted phenyl C=C, stretching), 1359 and 1172, (${SO}_{2}^{-}$, asymmetric and symmetric stretching), and 816 (C-H, out-of-plane bending). ^1^H NMR (400 MHz, DMSO-*d*_6_, ppm) δ 7.74–7.61 (m, 8H), 7.51–7.42 (m, 8H), 3.81 (d, J = 3.6 Hz, 8H), 2.43 (s, 12H). ^13^C NMR (400 MHz, DMSO-*d*_6_, ppm) δ 148.12 (C_4_), 146.36 (C1), 131.41 (C_2_), 128.33 (C_3_), 67.24 (C_6_), 42.94 (C_7_), and 21.32 (C_5_) ppm. MALDI-TOF MS (*m*/*z*): calculated for C_33_H_36_O_12_S_4_, 752.9; found, 776.2 for [M + Na]^+^.

### 3.3. Synthesis of Ethylenediamino-β-Cyclodextrin (EDA-β-CD)

A total of 10 g (7.76 mmoL) of TsO-β-CD was dissolved into anhydrous ethylenediamine (60 mL) in a three-necked flask under stirring. The mixed solution was heated up to 85 °C and further stirred for another 48 h. The reaction was terminated and the solution was concentrated by rotary evaporation. The mixed solution was added into 300 mL acetone and filtered. The precipitation was dissolved in water/methanol (3:1 *v*/*v*). (KBr, cm^−1^): 3200–3400 (O-H and N-H overlapping stretching), 2933 (-CH_2_-, stretching), 1413 (C-N, stretching), and 1160–1000 (C-O-C, stretching). ^1^H NMR (400 MHz, D_2_O, ppm) δ 5.04 (d, J = 3.8 Hz, H_1_), 3.90 (dd, J = 10.0, 9.0 Hz, ^1^H), 3.62–3.47 (m, H_2_-H_6_), 2.77–2.72 (m, H_7_-H_9_). ^13^C NMR (400 MHz, D_2_O, ppm) 101.92 (C_1_), 83.42 (C_4_), 81.45 (C_2_), 72.94 (C_3_), 72.37 (C_5_), 59.78 (C_6_), 51.11 (C_8_), 48.89 (C_7_), and 40.96 ppm (C_9_). MALDI-TOF MS (*m*/*z*): calculated for C_44_H_75_N_2_O_35_, 1192.1; found, 1215.2 for [M + Na]^+^.

### 3.4. Synthesis of Cyclodextrin Tetramer

EDA-β-CD (5.2 g, 4.4 mmoL) and pentaerythrityl tetratosylate (0.83 g, 1.1 mmoL) were dissolved into DMF (60 mL) in a three-necked flask under stirring, and then an appropriate amount of HCl was dropped into the above reaction mixture. Subsequently, the mixed solution was heated up to 85 °C and further stirred for 8 h. Next, the reaction was terminated and the solution was cooled to room temperature. In the end, the mixed solution was added into 300 mL absolute alcohol and filtered. IR (KBr, cm^−1^): 3409 (O-H and N-H, overlapping stretching), 2924 (-CH_2_-, stretching), 1407 (C-N, stretching), and 1150–1000 (1157, 1088, 1036) (C-O-C, stretching). ^1^H NMR (400 MHz, DMSO-*d*_6_, ppm) δ 5.68–5.74 (OH2, OH3), 4.83 (H_1_), 4.45 (OH6), 3.82–3.48 (H_3_, H_5_, and H_6_), 3.46–3.12 (H_2_ and H_4_), 2.89 (H_8_), 2.73 (H_11_), 2.31 (H_10_), 2.19, (H_9_), 2.12 (H_7_). ^13^C NMR (400 MHz, DMSO-*d*_6_, ppm) 102.55 (C_1_), 84.51 (C_2_), 83.42 (C_4_), 74.07–71.98 (C_3_ and C_5_), 60.57 (C_6_), 49.91–48.54 (C_7_-C_9_, C11), and 19.01 (C_10_). MALDI-TOF MS (*m*/*z*): calculated for C_181_H_308_N_8_O_136_, 4768.1; found, 4790.6 for [M + Na]^+^.

### 3.5. Synthesis of Cyclodextrin-Based Bispolyrotaxane (CBBP)

In detail, amino-terminated PPG (PPG-NH_2_, 0.08 g, 0.04 mmoL) was added dropwise to a saturated aqueous solution (10 mL) of the cyclodextrin tetramer (3.82 g, 0.8 mmoL) with a strong base (NaOH, 10%) at room temperature. The mixture was stirred for 24 h, and then superfluous 2, 4-dinitrofluorobenzene (DFB, 20 μL, 0.16 mmoL) was dropped into the reaction system and vigorously stirred for another 36 h. The reaction was terminated and the reaction solution was neutralized by the addition of HNO_3_ (20%). After concentration, the mixture was poured into 300 mL of ethanol, and the fulvous precipitate was collected by filtration. IR (KBr, cm^−1^): 3423 (O-H and N-H, overlapping stretching), 2925 (-CH_2_-, stretching), 1530 and 1383 (-NO_2_, stretching), 1412 (C-N), 1150–1000 (1155, 1082, 1034) (C-O-C, stretching), and 940 (pyranose ring, stretching) (Figure S13). ^1^H NMR (400 MHz, DMSO-*d_6_*, ppm) δ 8.92–7.43 (H_7_-H_9_ of DFB), 5.95–5.59 (OH2 and OH3 of β-CD), 4.86 (H_1_ of β-CD), 4.46 (OH6 of β-CD), 3.67–3.35 (H_2_-H_6_ of β-CD; -CH_2_CHO- of PPG-NH_2_), and 1.08 (H_10_, -CH_3_ of PPG-NH_2_) (Figure S14).

The threading of a polymer axle (DFB-PPG-NH_2_) into the cavities of CD rings was characterized by ^1^H NMR and 2D ROESY NMR. There was an upfield shift of Δδ = 0.078–0.186 ppm for the PPG-NH_2_ methyl protons (H_10_) (Figure S15), while the H_3_/H_5_ protons of the β-CD units in the CBBP shifted downfield by Δδ = 0.017 ppm, respectively (Figure S16). Furthermore, 2D ROESY NMR (400 MHz, DMSO-*d*_6_, ppm): a pronounced cross-peak (labeled A) was detected, which indicated a correlation between the methyl (H_10_) protons of PPG-NH_2_ and β-CD moiety protons (H_3_, H_5_) in the CBBP (Figure S17).

GPC (average molecular weight): A unimodal molecular weight distribution was observed in the GPC curves, indicating that no homopolymers existed in the CBBP. Accordingly, the average molecular weight of the CBBP was measured to be ~4.3 × 10^4^ g·mol^−1^. Analysis of the average molecular weight of the CBBP indicates that it is a homogeneous polymer rather than a polydisperse aggregate (Figure S18).

### 3.6. Synthesis of Cyclodextrin-Based Bimolecular Tubes (CBBTs)

The CBBT was prepared via a modified method reported. Briefly, the CBBP (1.4 g) was added to a 10% aqueous NaOH solution (10 mL), and then the epichlorohydrin (205 μL, 2.62 mmoL) was dropped into the above solution. The reaction proceeded for 36 h at room temperature and was then neutralized by the addition of HNO_3_ (20%). Subsequently, the mixed solution was dialyzed (MWCO 1000) for 3 days and concentrated by rotary evaporation. Finally, the mixed solution was added into 300 mL ethanol, and then the deep yellow precipitate was filtered. In order to remove DFB-PPG, the prepared sample (2 g) was added to a 25% aqueous NaOH solution (20 mL), and the mixture was stirred for 24 h at 45 °C. At the end of the reaction, the mixed solution was cooled and then neutralized with HNO_3_ (20%). In this case, the mixed solution was added into 300 mL ethanol to give a deep yellow precipitate. After collection by filtration, the precipitate was washed thoroughly three times with acetone.

### 3.7. Synthesis of PANI/CBBT Bispolypseudorotaxane

Firstly, aniline (93 mg, 1.0 mmoL) was introduced into an aqueous solution (50 mL) of the bis-molecular tube (1.5 g, 1.0 mmoL). The pH value of the mixed solution was adjusted to 2 using hydrochloric acid. Then, the mixture was stirred at room temperature for 10 h. Ultimately, ammonium persulfate (228 mg, 1.0 mmoL) was added, and stirring was maintained at room temperature for another 10 h. Finally, the dark green precipitate that formed was collected via filtration, thoroughly washed with water, and dried under vacuum.

## 4. Conclusions

Overall, we have developed an efficient strategy for the construction of PANI/CBBT bispolypseudorotaxane. The key to this strategy lies in the initial formation of a stable bispseudorotaxane precursor, achieved by threading aniline monomers into the nanoconfined spaces of the CBBT. This pre-assembly step was critical, as it preorganized the anilines in a linear array and prevented their uncontrolled aggregation in the bulk solution. Upon the addition of an oxidant, the confined aniline monomers simultaneously underwent oxidative polymerization, resulting directly in the formation of a bispolypseudorotaxane structure where the conjugated PANI backbone was permanently encapsulated by the CBBT.

## Data Availability

The data supporting the findings of this study are available as follows: all raw data, including experimental details, spectroscopic figures, and electron microscope images, are provided within the manuscript and its Supporting Information.

## Supplementary Materials

The following supporting information can be downloaded at: https://www.mdpi.com/article/10.3390/molecules31183353/s1. Figure S1. FT-IR spectrum of pentaerythrityl tetratosylate. Figure S2. ^1^H NMR spectra of pentaerythrityl tetratosylate in DMSO-*d_6_* at 25 °C. Figure S3. ^13^C NMR spectra of pentaerythrityl tetratosylate in DMSO-*d_6_* at 25 °C. Figure S4. MALDI-TOF MS of pentaerythrityl tetratosylate. Figure S5. FT-IR spectrum of EDA-*β*-CD. Figure S6. ^1^H NMR spectra of EDA-*β*-CD in DMSO-*d_6_* at 25 °C. Figure S7. ^13^C NMR spectra of EDA-*β*-CD in DMSO-*d_6_* at 25 °C. Figure S8. MALDI-TOF MS of EDA-*β*-CD. Figure S9. FT-IR spectrum of cyclodextrin tetramer. Figure S10. ^1^H NMR spectrum of cyclodextrin tetramer. Figure S11. ^13^C NMR spectrum of cyclodextrin tetramer. Figure S12. MALDI-TOF MS of cyclodextrin tetramer. Figure S13. FT-IR spectrum of CBBP. Figure S14. ^1^H NMR spectrum of CBBP. Figure S15. The interaction between PPG-NH_2_ and cyclodextrin tetramer. Figure S16. The expanded region of the ^1^H NMR spectrum of PPG-NH_2_ and CBBP. Figure S17. Sectional 2D ROESY NMR spectrum of PPG-NH_2_ (5.0 × 10^−3^ mmol) and cyclodextrin tetramer (5.0 × 10^−3^ mmol) with a mixing time of 5 min in DMSO-*d*_6_ at 25 °C. Figure S18. The average molecular weight of CBBP. Figure S19. FT-IR spectrum of CBBT. Figure S20. XRD pattern of TsO-β-CD (a) EDA-β-CD, cyclodextrin tetramer, CBBP, and CBBT (b). Figure S21. WAXS pattern of CBBT. Figure S22. Expanded region of the ^1^H NMR spectrum of CBBT and aniline/CBBT complex. Figure S23. (a) Expanded region of the ^1^H NMR spectrum of CBBT, PANI, and PANI/CBBT bispolypseudorotaxane. (b,c) Schematic diagram of the structure of *β*-CD and PANI. Figure S24. XRD spectra of CBBT, PANI, and PANI/CBBT bispolypseudorotaxane.
