# Supplementary material for: Confined Oxidative Polymerization of Preorganized Aniline Within Cyclodextrin-Based Bimolecular Tube Nanochannels for the Synthesis of Bispolypseudorotaxanes
Source: Molecules. 2026 Sep 21;31(18):3353. doi: 10.3390/molecules31183353 (PMC13610029; doi:10.3390/molecules31183353)
Supplement: Supplementary file 1 [file molecules-31-03353-s001.zip › molecules-4533928-supplementary.pdf]

Confined oxidative polymerization of preorganized aniline within cyclodextrin-based bimolecular tubes nanochannels for the synthesis of bispolypseudorotaxanes

Min Deng[1], Xuze Hu[1, 2,*], Yibing Liu[1], Yuqiang Cheng[1], Donghua Lai[1], Lu Huang[1], Xinli Mou[1], Junsheng Qi[1], Changjun Zou[3]

[1]*Chongqing Key Laboratory of Water Environmental Evolution and Pollution Control in Three Gorges Reservoir, School of Environmental and Chemical Engineering, Chongqing Sanxia University of Science and Technology, Chongqing 404100, P.R. China. E-mail: hj127@163.com*

[2]*Material Corrosion and Protection Key Laboratory of Sichuan province, Sichuan University of Science & Engineering, Zigong 643000, P.R. China. E-mail: hj127@163.com*

[3]*School of chemistry and chemical Engineering, Southwest Petroleum University, Chengdu 610500, P.R. China. E-mail: changjunzou@126.com*

Supplemental Figures

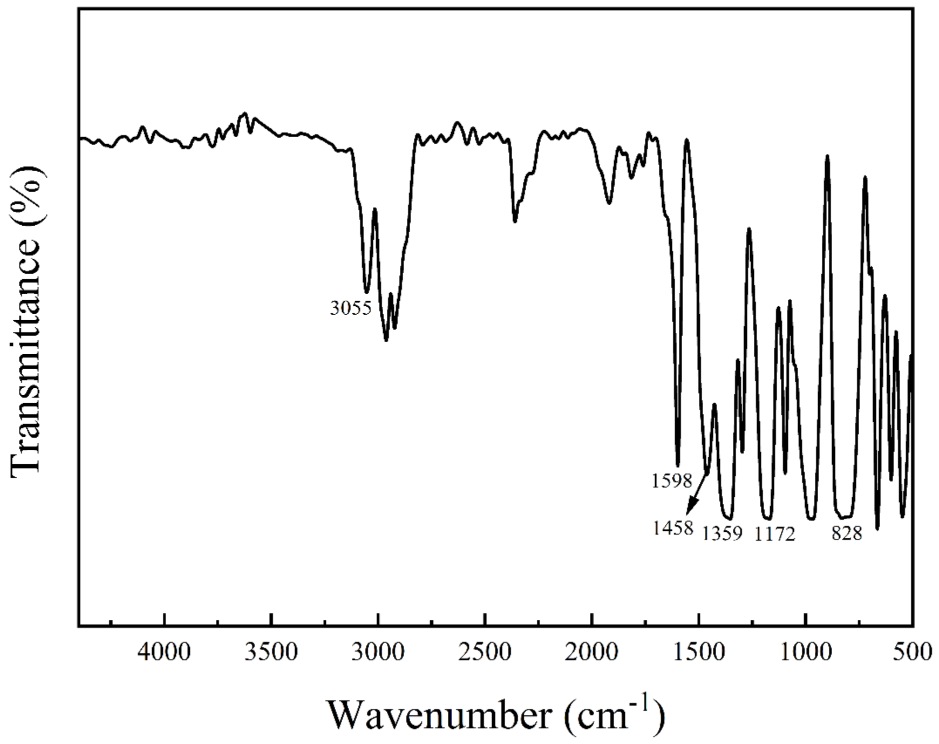

Figure S1. FT-IR spectrum of pentaerythrityl tetratosylate.

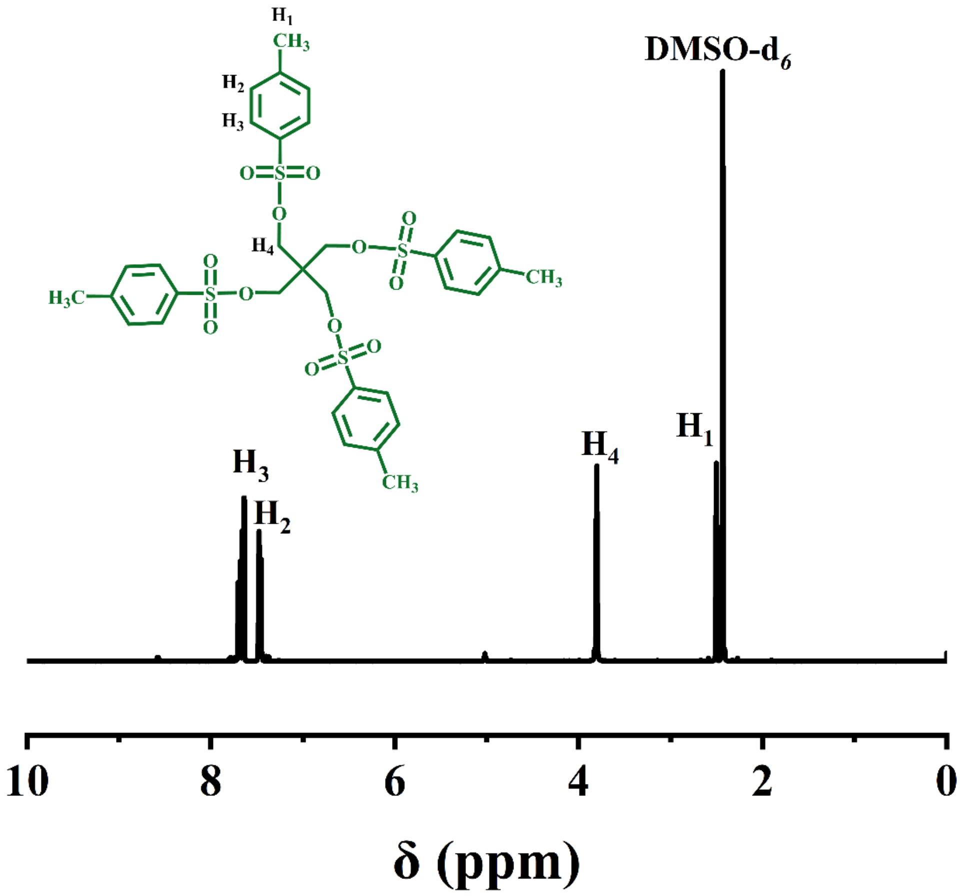

Figure S2. ^{1}H NMR spectra of pentaerythrityl tetratosylate in DMSO-d_6 at 25 °C.

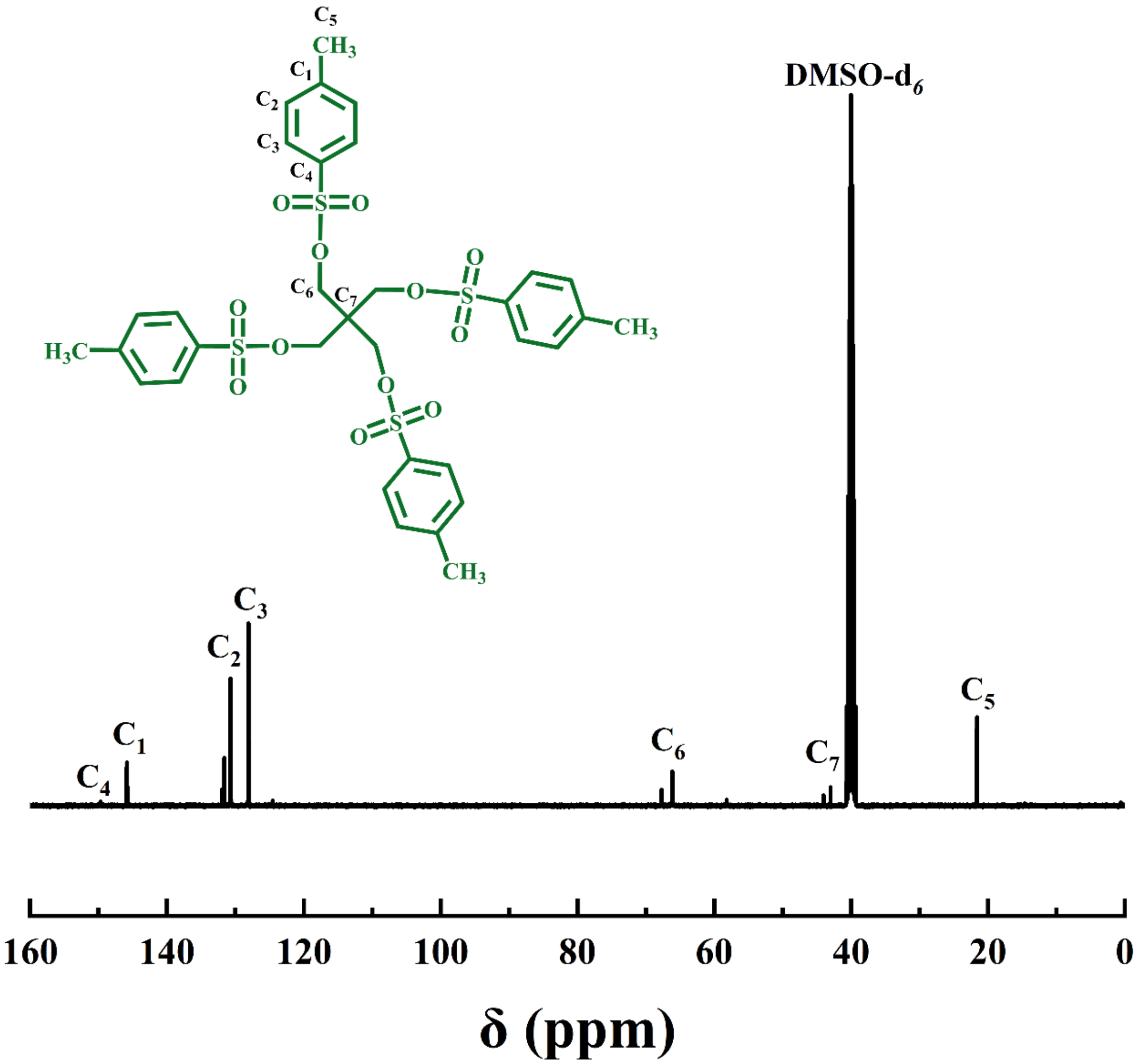

Figure S3. ^{13}C NMR spectra of pentaerythrityl tetratosylate in DMSO-d_6 at 25 °C.

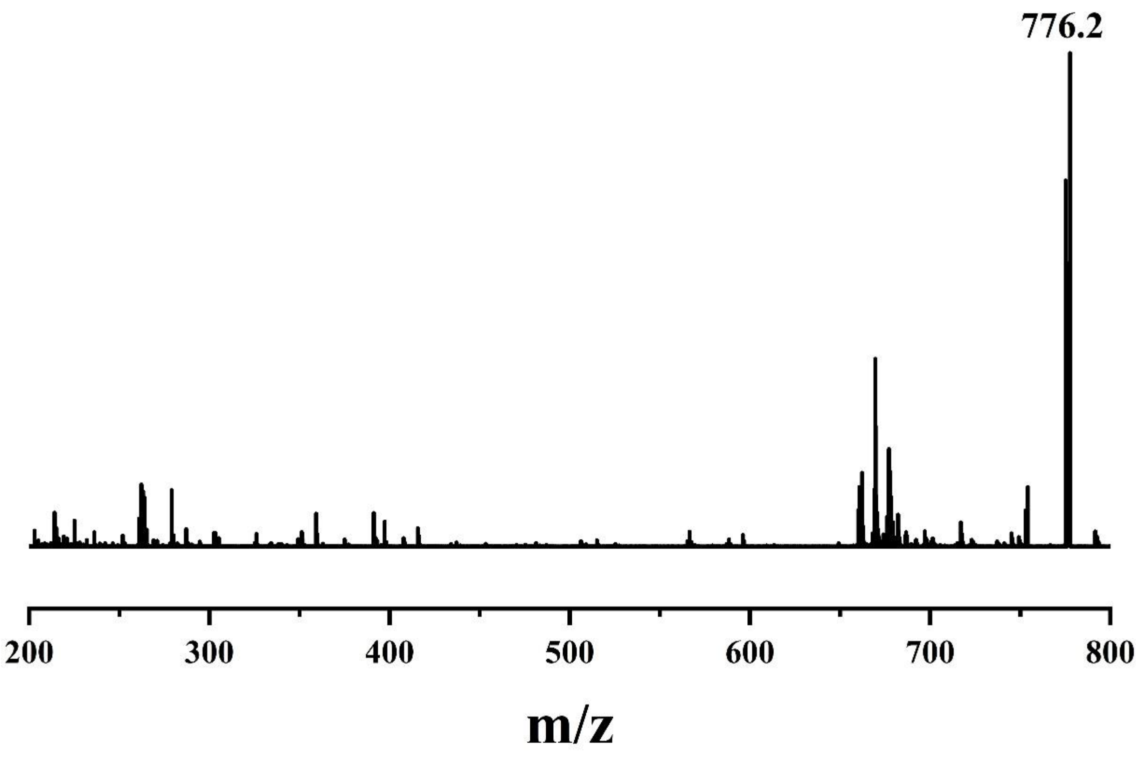

Figure S4. MALDI-TOF MS of pentaerythrityl tetratosylate.

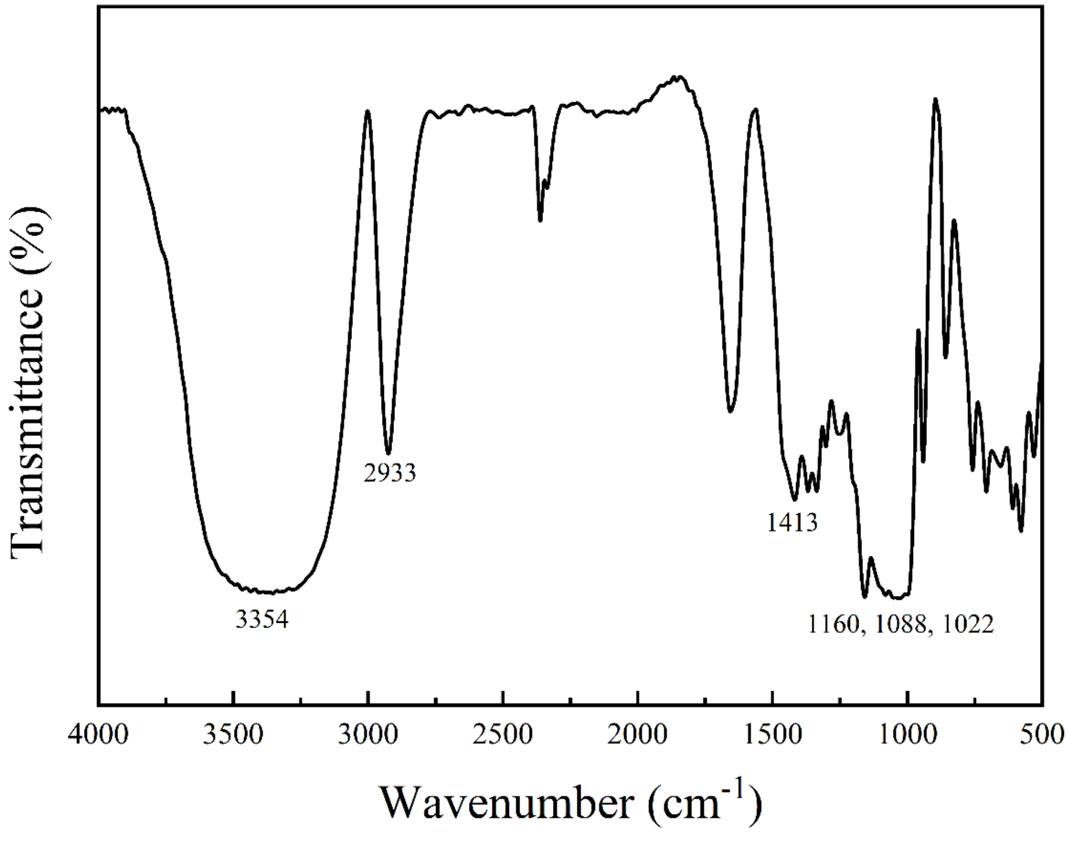

Figure S5. FT-IR spectrum of EDA-*β*-CD.

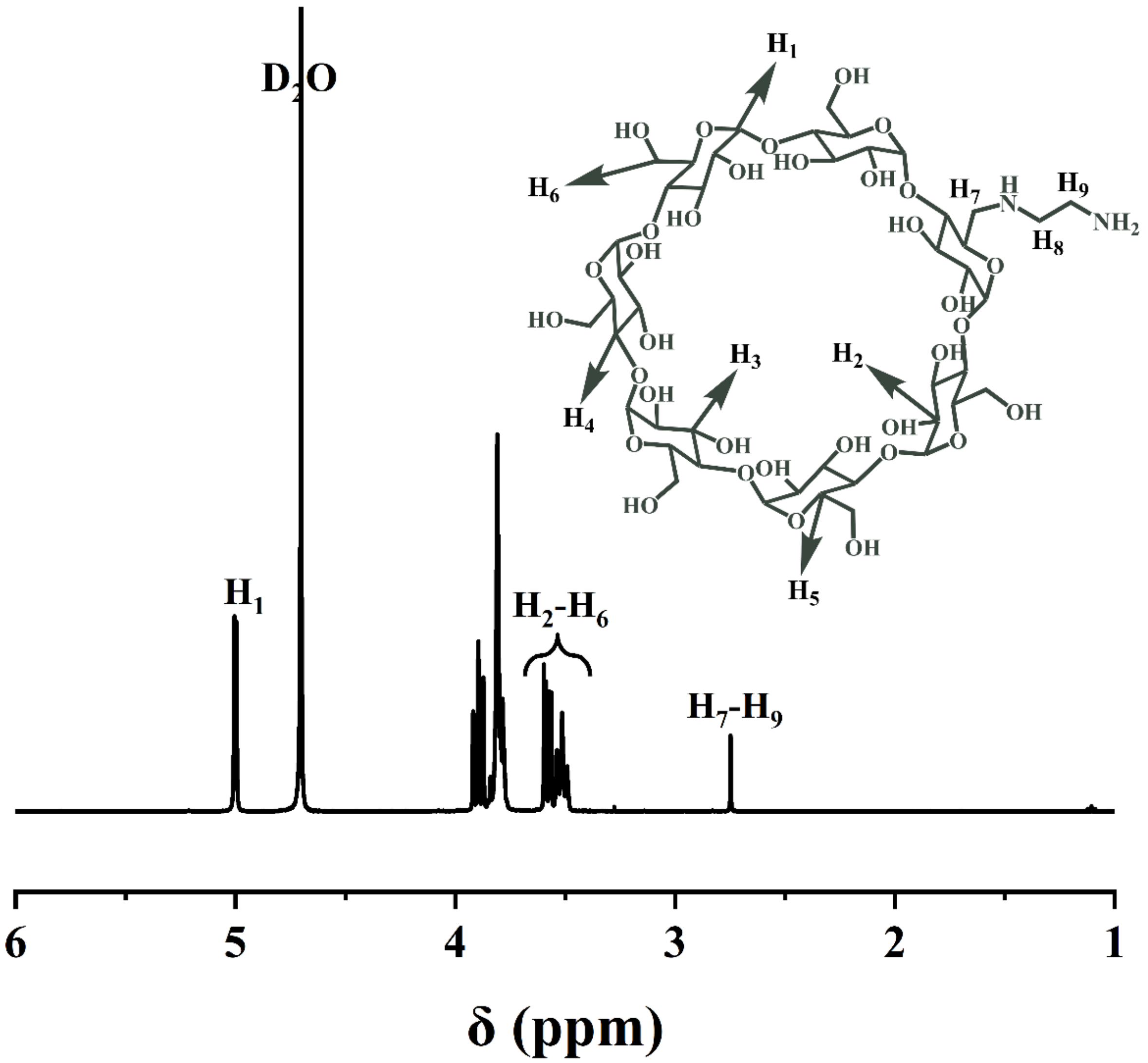

Figure S6. ^{1}H NMR spectra of EDA-*β*-CD in DMSO-*d*$_6$ at 25 °C.

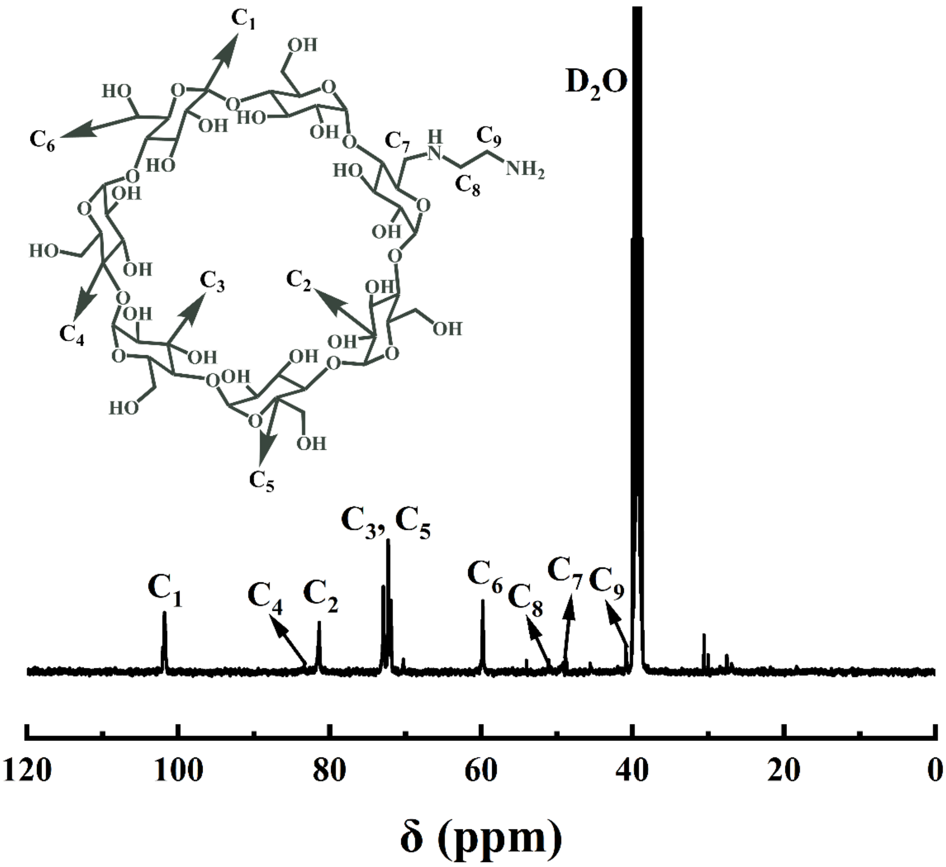

Figure S7. ^{13}C NMR spectra of EDA-*β*-CD in DMSO-*d*$_6$ at 25 °C.

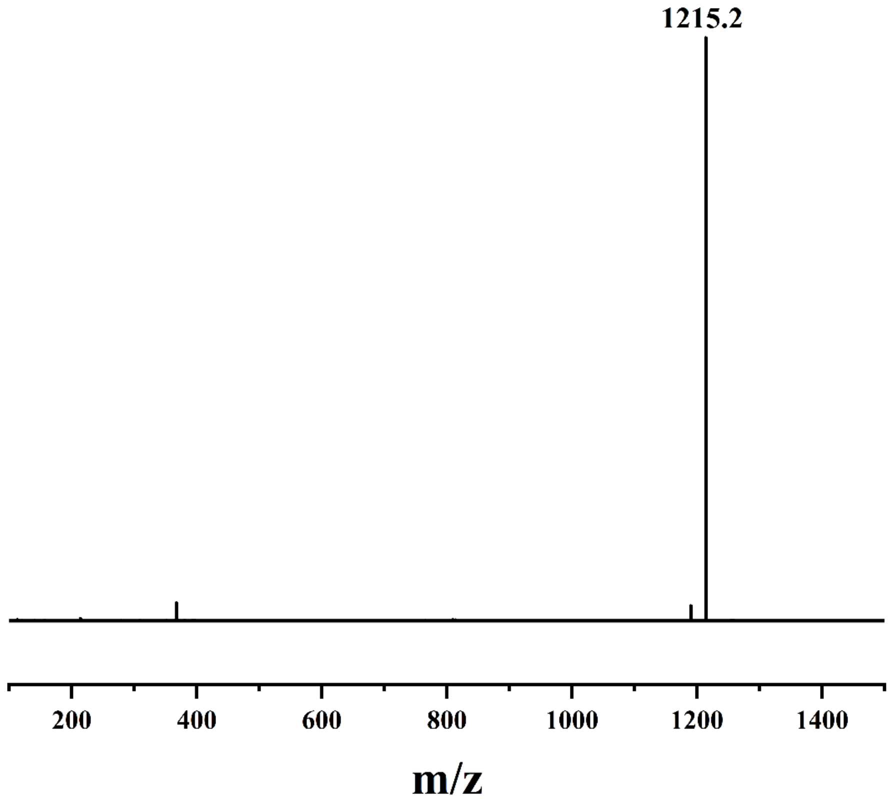

Figure S8. MALDI-TOF MS of EDA-*β*-CD.

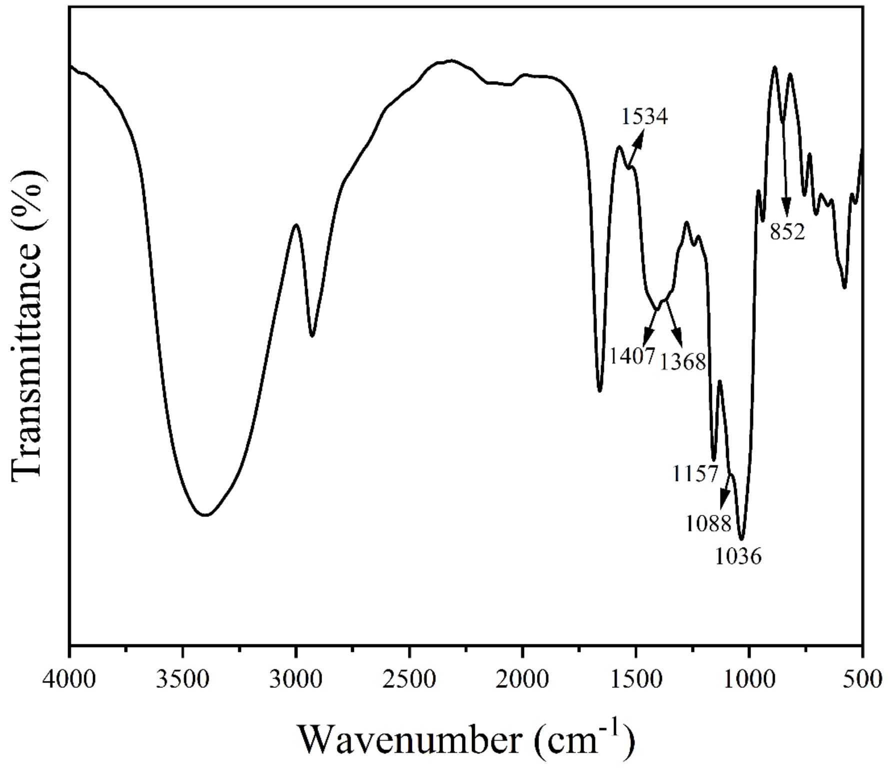

Figure S9. FT-IR spectrum of cyclodextrin tetramer.

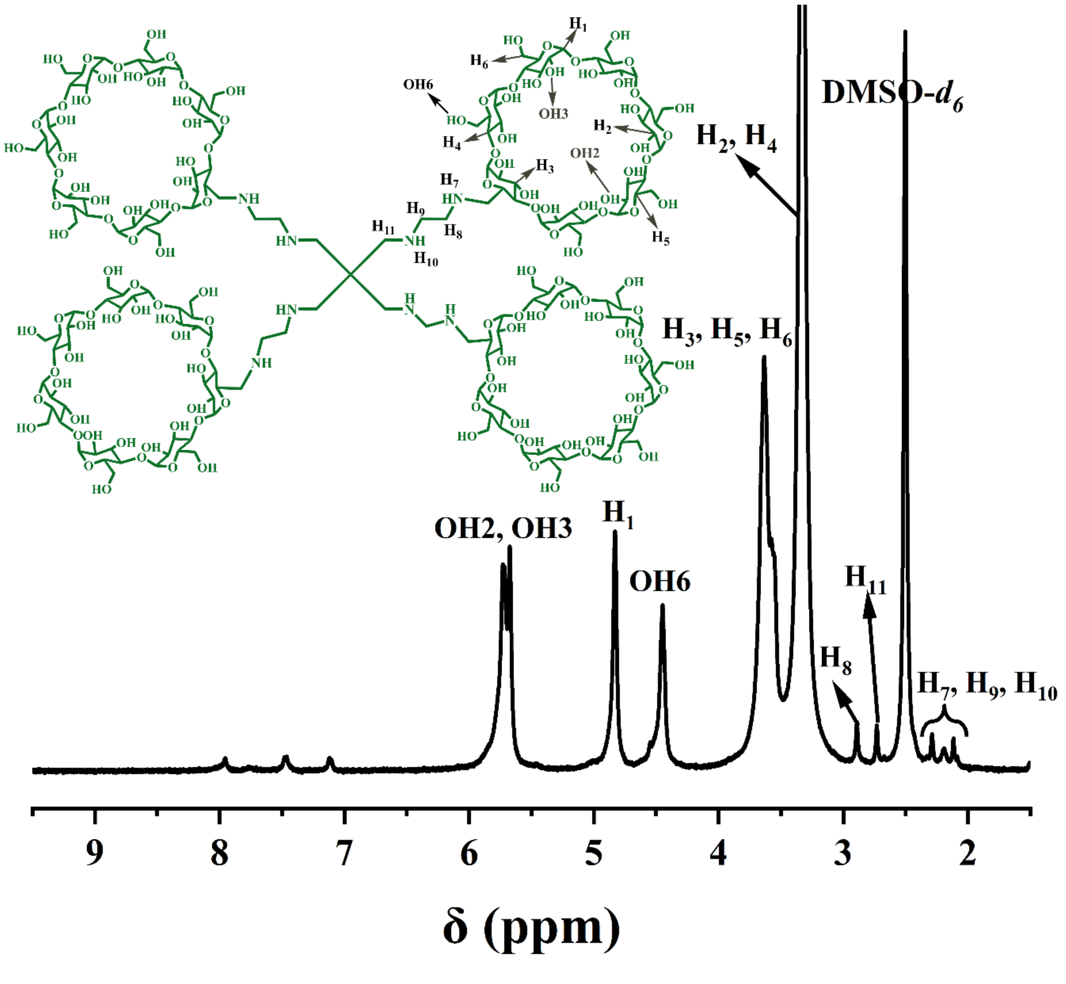

Figure S10. ^{1}H NMR spectrum of cyclodextrin tetramer.

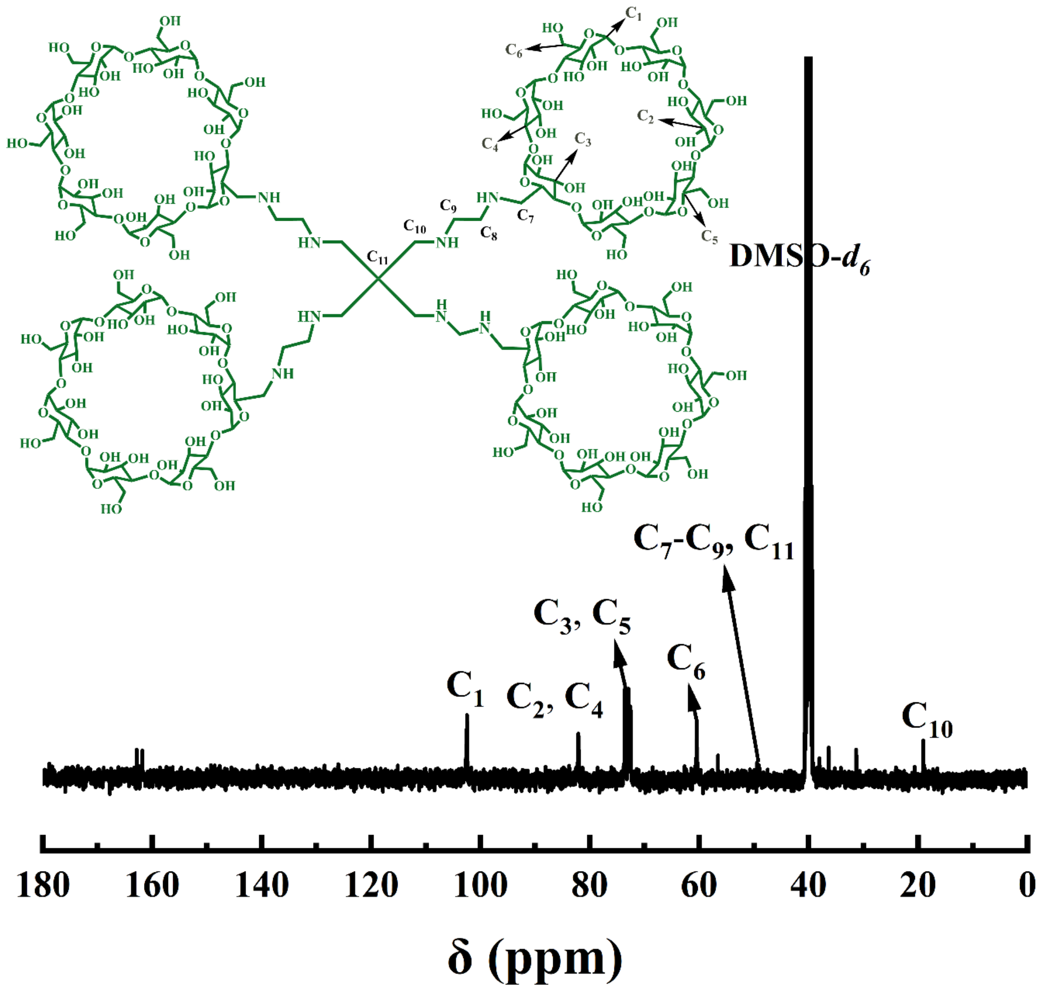

Figure S11. ^{13}C NMR spectrum of cyclodextrin tetramer.

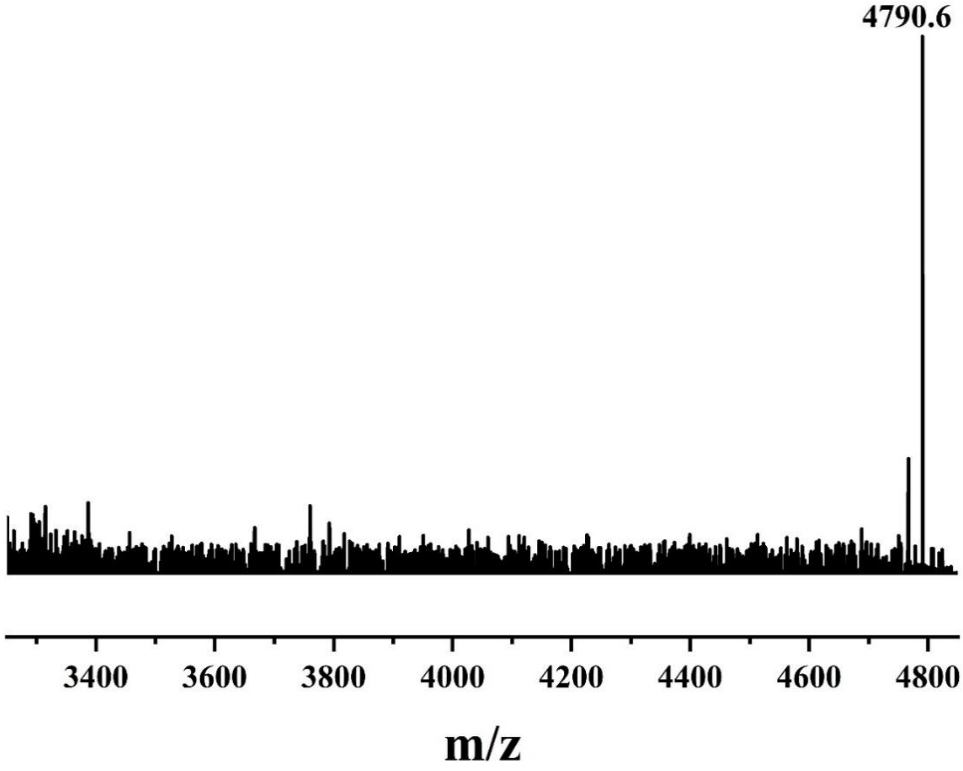

Figure S12. MALDI-TOF MS of cyclodextrin tetramer.

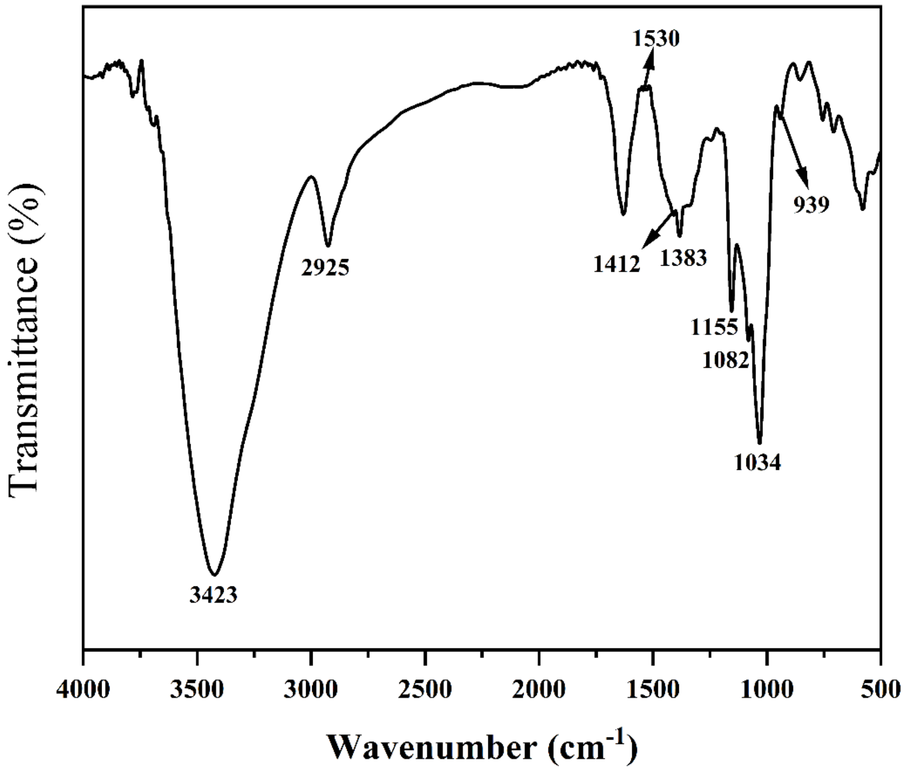

Figure S13. FT-IR spectrum of CBBP.

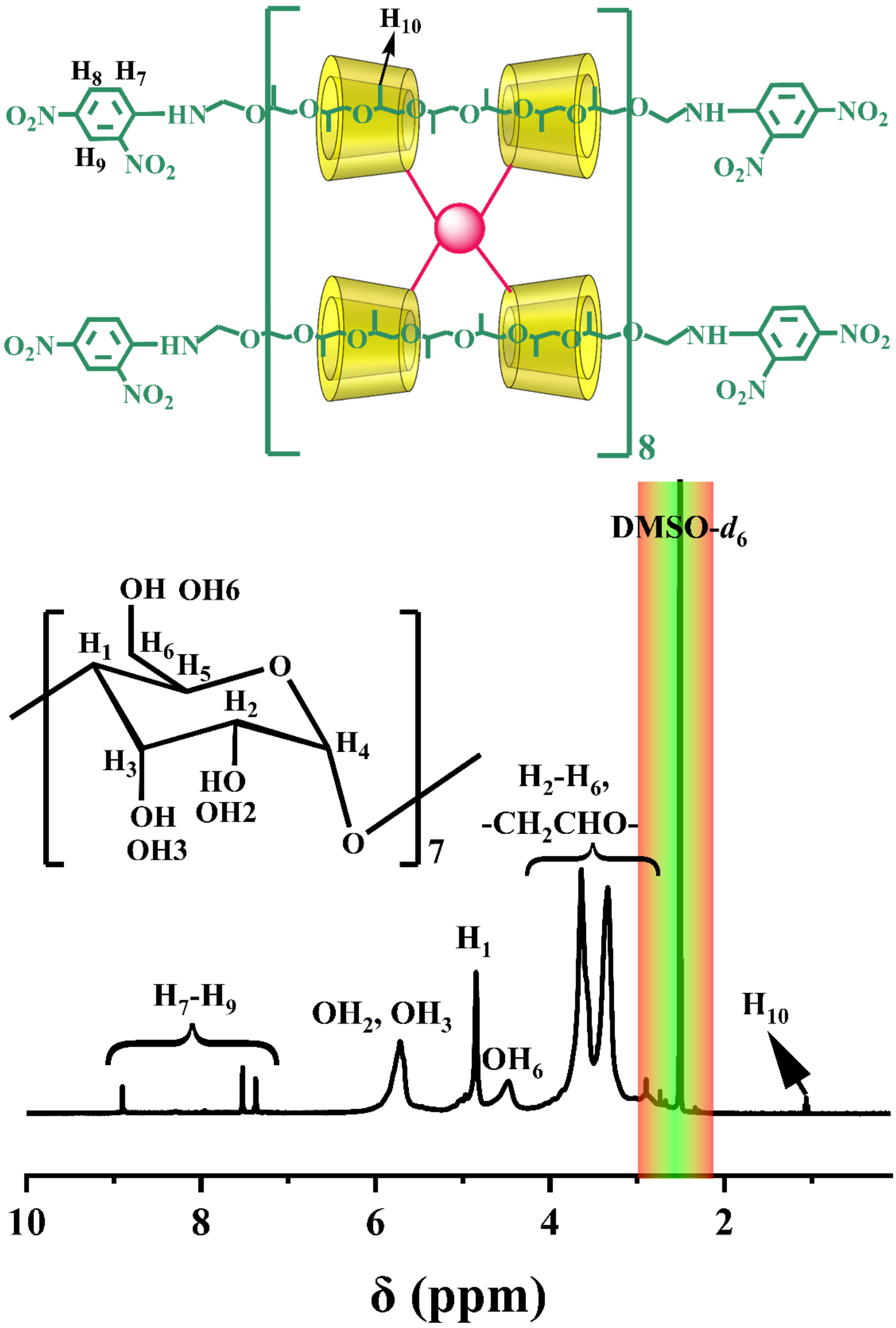

Figure S14. ^{1}H NMR spectrum of CBBP.

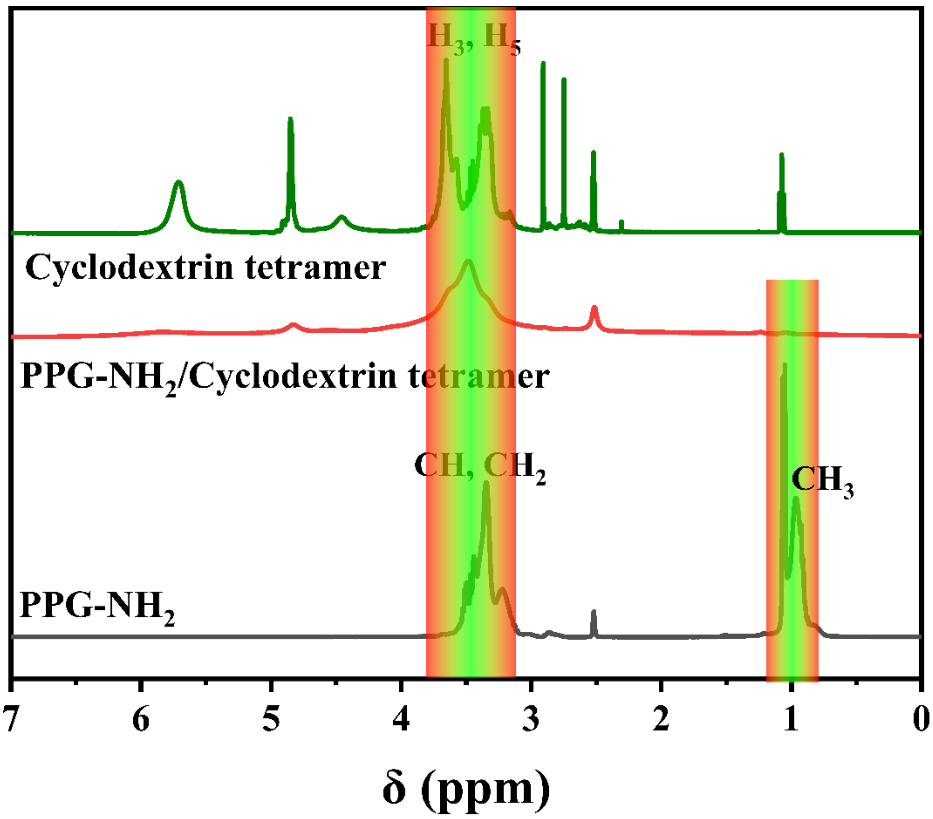

Figure S15. The interaction between PPG-NH_2 and cyclodextrin tetramer.

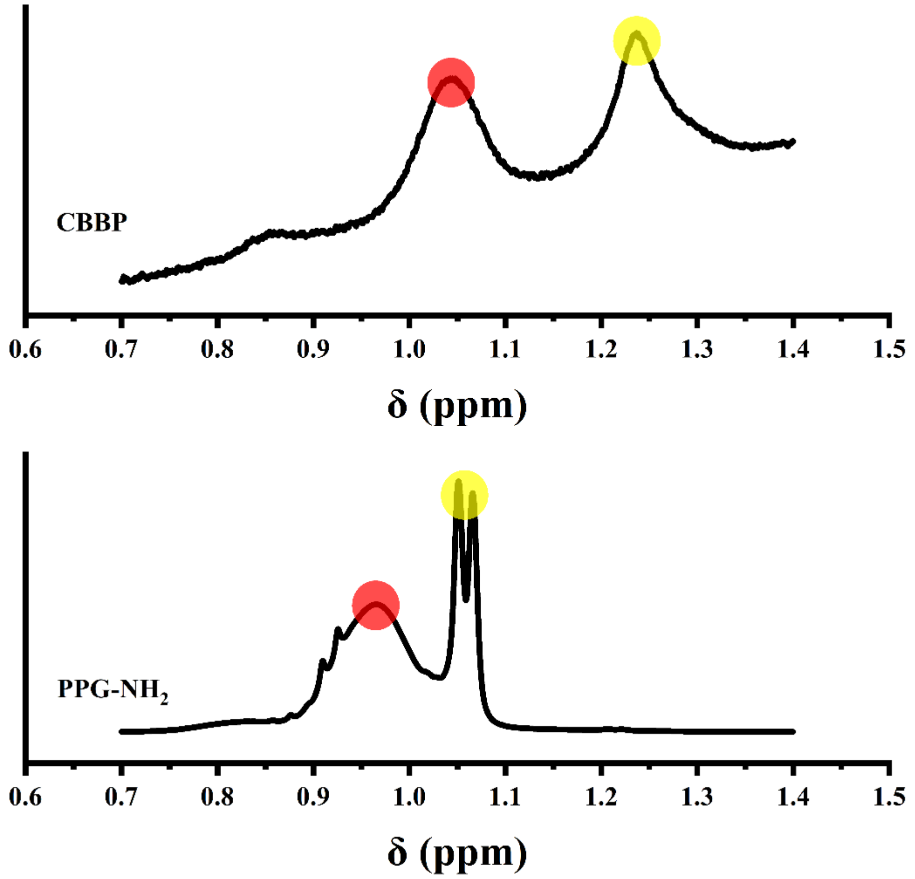

Figure S16. The expanded region of the ^{1}H NMR spectrum of PPG-NH_2 and CBBP.

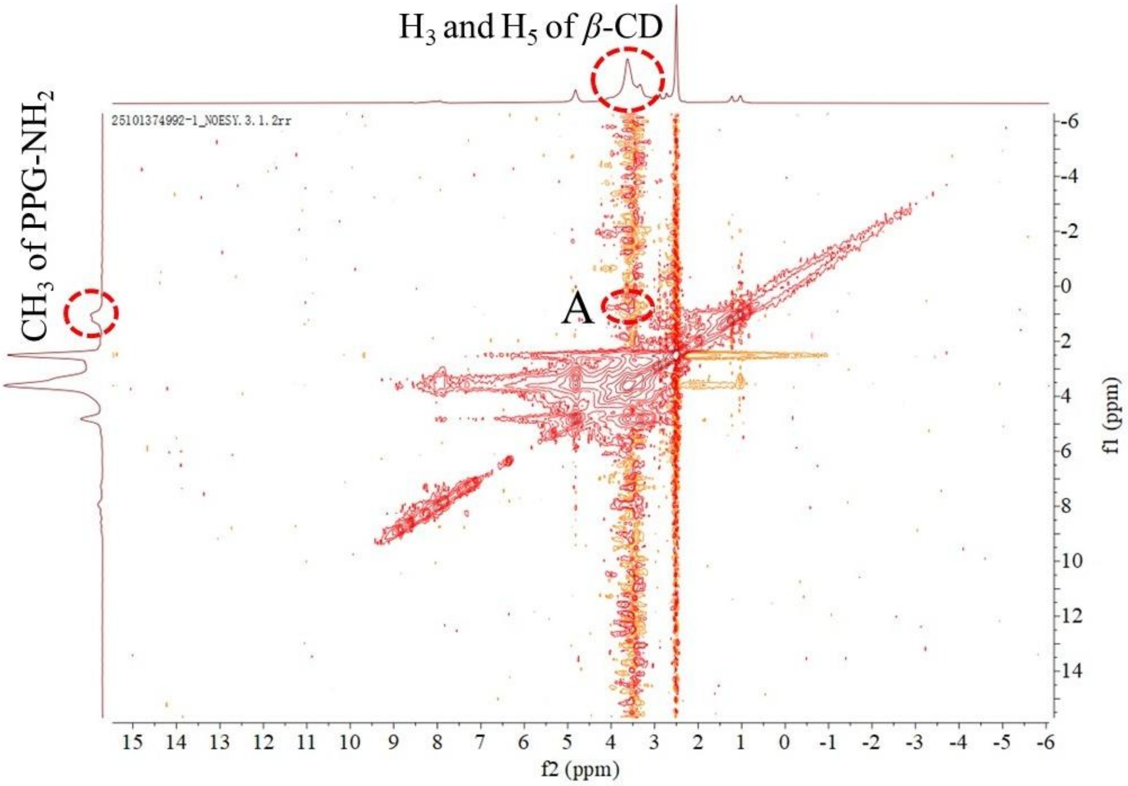

Figure S17. Sectional 2D ROESY NMR spectrum of PPG-NH_2 (5.0×10^{-3} mmol) and cyclodextrin tetramer (5.0×10^{-3} mmol) with a mixing time of 5 min in DMSO-d_6 at 25 °C.

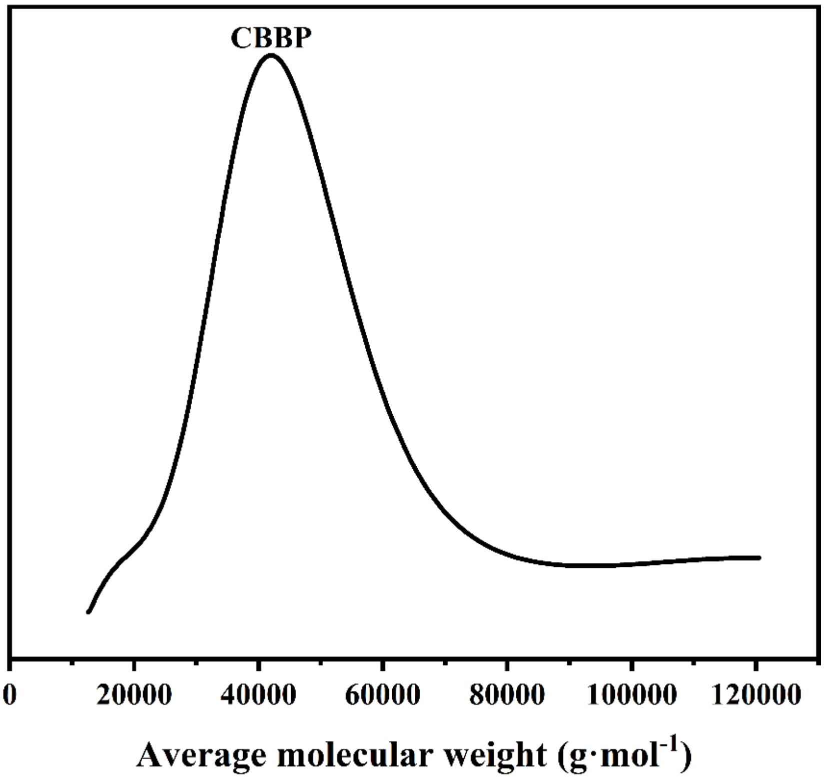

Figure S18. The average molecular weight of CBBP.

3. Supplemental Figures

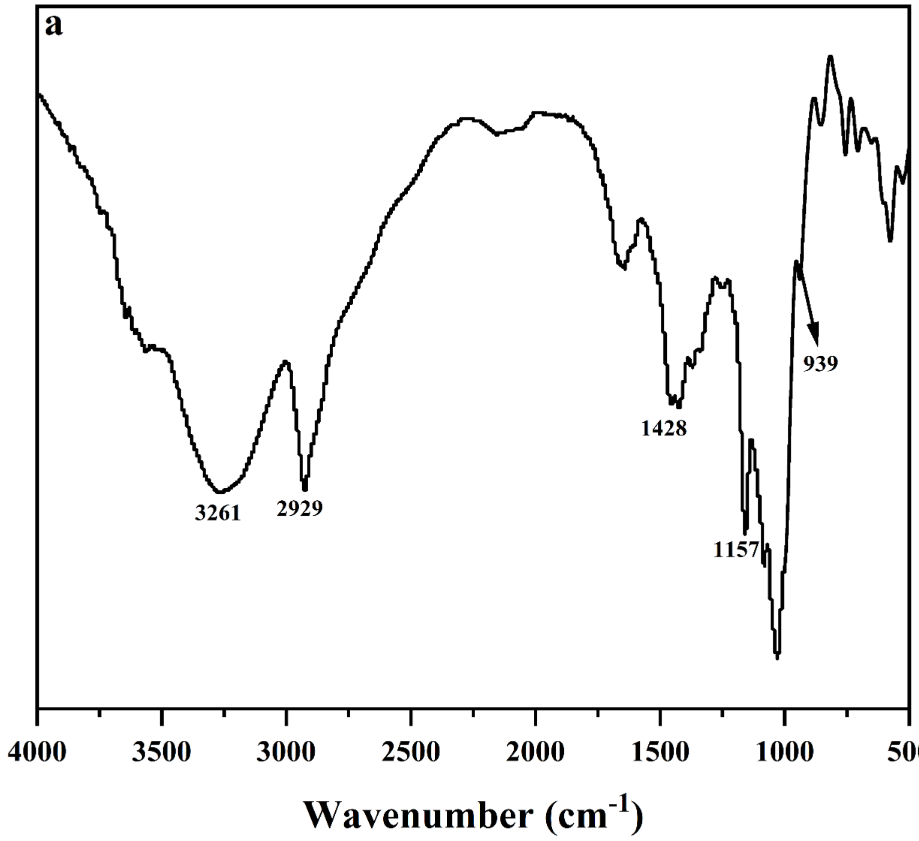

Figure S19. FT-IR spectrum of CBBT.

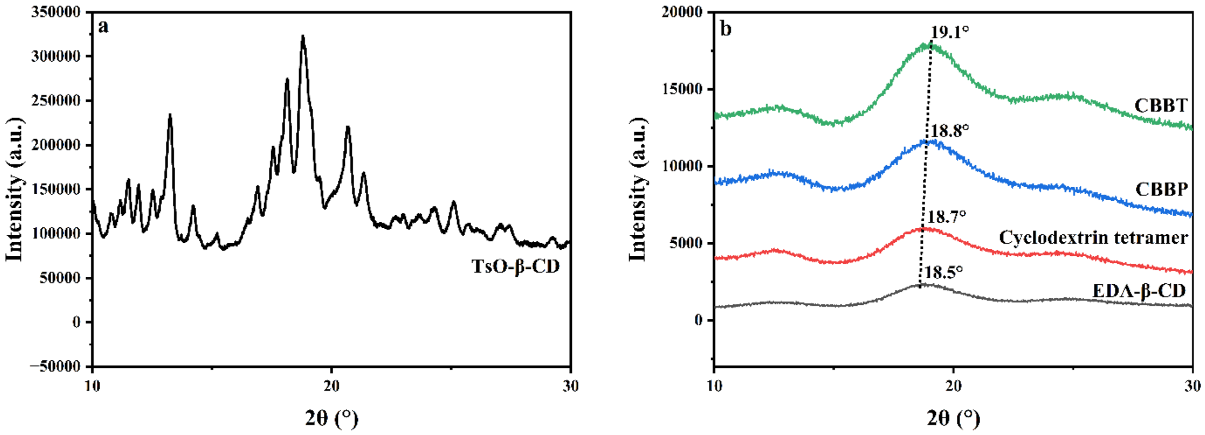

Figure S20. XRD pattern of TsO-β-CD (a) EDA-β-CD, cyclodextrin tetramer, CBBP, and CBBT(b).

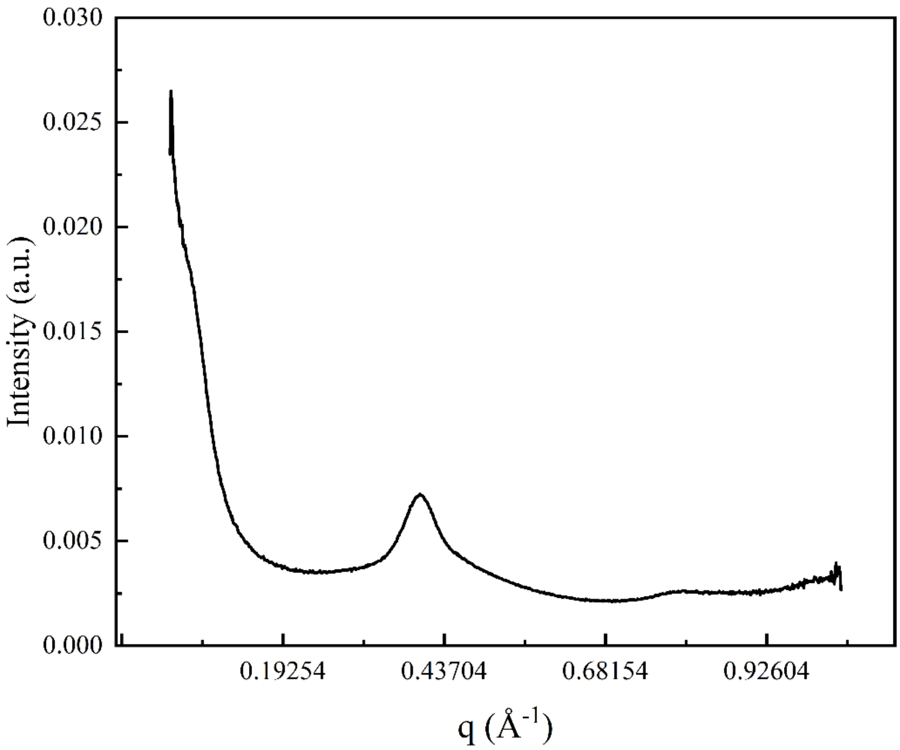

Figure S21. WAXS pattern of CBBT.

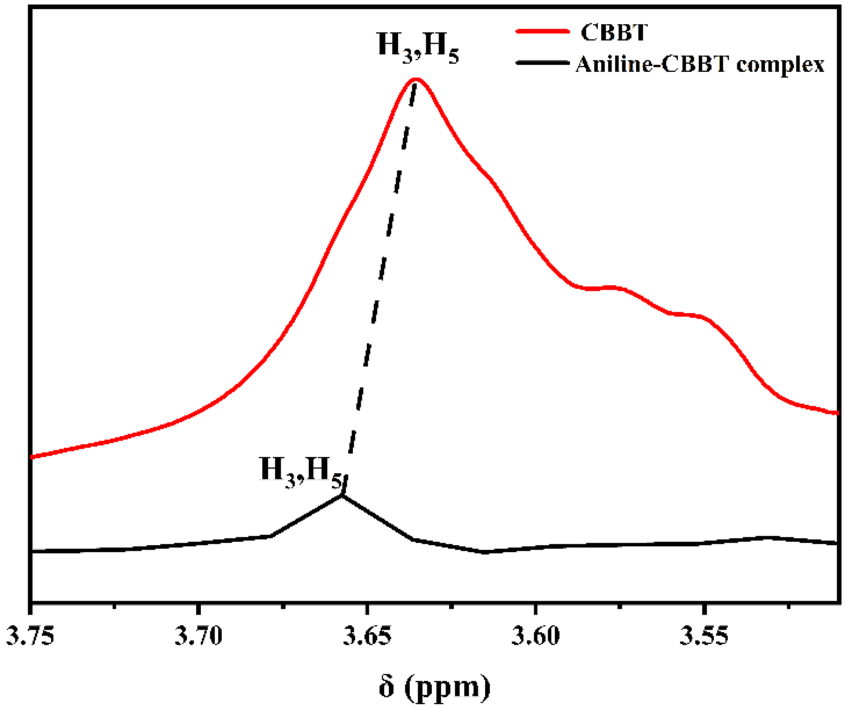

Figure S22. Expanded region of the ^{1}H NMR spectrum of CBBT and aniline/CBBT complex.

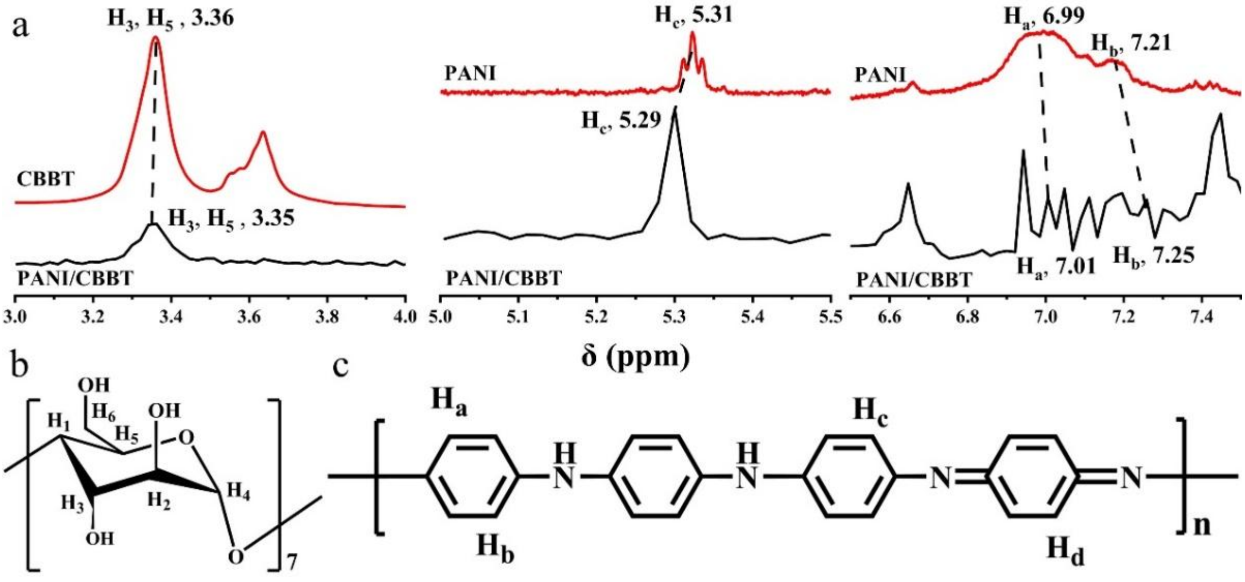

Figure S23. (a) Expanded region of the ^{1}H NMR spectrum of CBBT, PANI, and PANI/CBBT bispolypseudorotaxane. (b, c) Schematic diagram of the structure of *β*-CD and PANI.

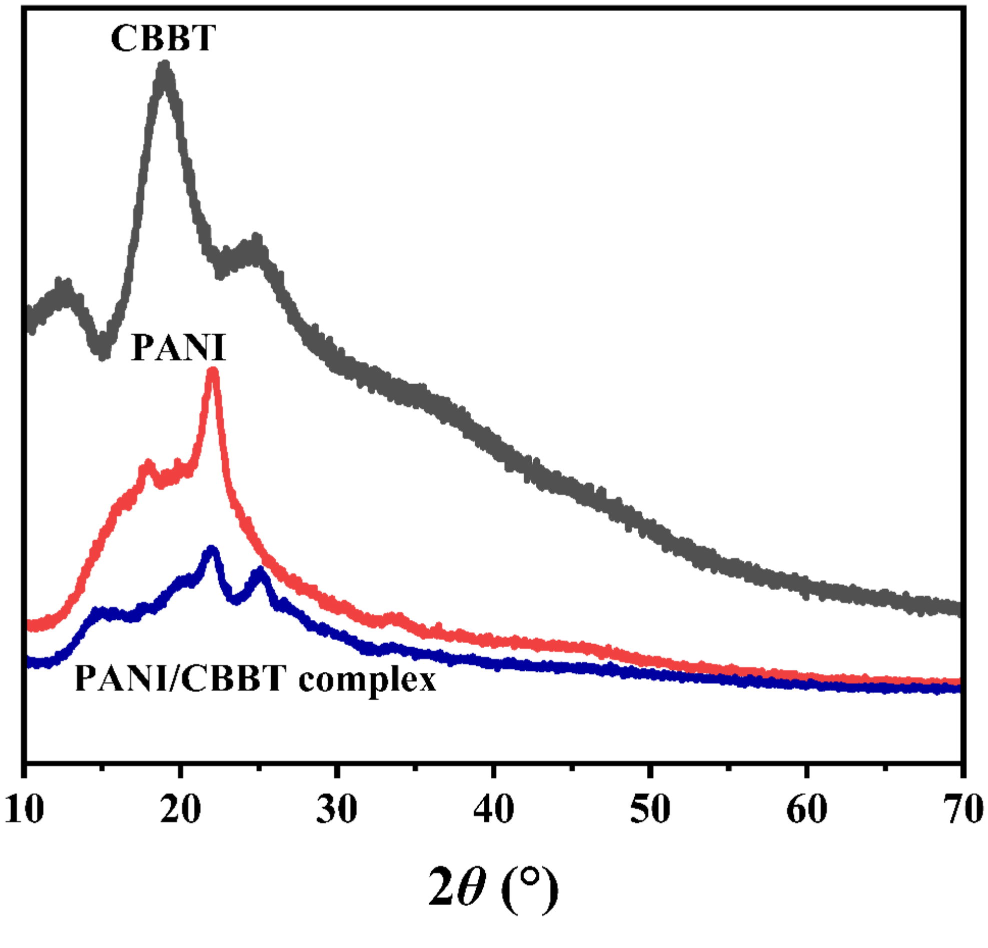

Figure S24. XRD spectra of CBBT, PANI, and PANI/CBBT bispolypseudorotaxane.
